# High Entanglement in Hydrogels: From Polymer Physics to Robust Mechanics

**DOI:** 10.1021/polymscitech.6c00001

**Published:** 2026-03-27

**Authors:** Yan Zhang, Hui Lou, Chunlei Yan, Qian Zhang, Yafei Wang, Yongjun Zhang

**Affiliations:** † State Key Laboratory of Separation Membranes and Membrane Processes, School of Chemistry, 47847 Tiangong University, Tianjin 300387, China; ‡ School of Materials Science and Engineering, Tiangong University, Tianjin 300387, China; § School of Pharmaceutical Sciences, Tiangong University, Tianjin 300387, China; ∥ Cangzhou Institute of Tiangong University, Cangzhou 300387, China

**Keywords:** chain entanglement, entangled hydrogel, topological constraint, swollen polymer networks, rheology, mechanical properties

## Abstract

Entangled hydrogels offer a strategy to address the mechanical limitations of swollen networks, including conflicts between stiffness and toughness and between water content and robustness. In these systems, dense topological constraints generated by chain interpenetration act as dynamic crosslinks that complement covalent junctions, promoting stress transfer, recoverable energy dissipation, and improved fatigue and anti-swelling performance. This review first outlines the polymer-physics basis of entanglement, emphasizing tube and reptation concepts, entanglement molecular weight, and entanglement density, and the respective contributions of chemical crosslinks and entanglements to the elasticity of hydrogels. It then analyzes key factors governing the degree and stability of entanglement, including concentration, architecture and flexibility of chains, solvent conditions, and processing history. Experimental methods for probing entangled networks are summarized, covering mechanical and rheological testing together with scattering, imaging, and spectroscopic techniques that access structure and dynamics over different length and time scales. The role of entanglement in the design of robust hydrogels is discussed, highlighting how linking polymer-physics descriptors to network design has enabled robust performance in applications such as wound repair and adhesive biomedical patches, high-deformation flexible sensors, hydrogel electrolytes for energy devices, and mechanically stable environmental remediation materials. Finally, the review highlights current challenges associated with swollen-state entanglement physics, long-term durability and environmental stability, and quantitative structure–property relationships needed for predictable design and control of entangled hydrogel networks.

## Graphical abstract

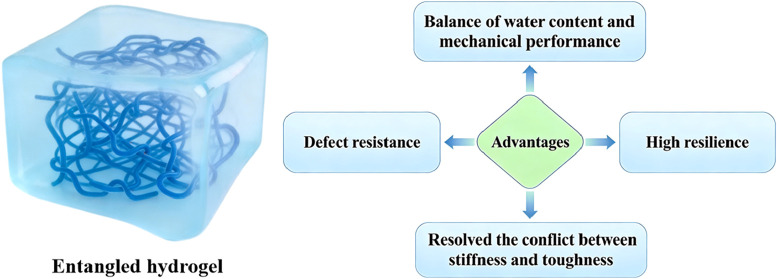

## 1 Introduction

Hydrogel is a polymer network system with hydrophilic, three-dimensional crosslinked structure. It can rapidly swell in water and retain a significant amount of water without dissolving.^1,2^ The formation of hydrogel occurs when water-soluble or hydrophilic polymers undergo chemical or physical crosslinking, creating a three-dimensional network structure in which hydrophilic groups interact with water molecules. This material possesses the solid-like ability to retain a defined shape and volume, while simultaneously exhibiting liquid-like characteristics that allow solutes to diffuse or permeate within its structure.^3^

Conventional hydrogels often suffer from inadequate mechanical properties, particularly when in their highly swollen state. This mechanical fragility, coupled with a lack of control over their swelling behavior, limits their practical applications (Figure 1a). Numerous studies have demonstrated that the irregularity within the hydrogel network is a critical factor contributing to its inadequate mechanical strength.^4^ When subjected to external stress, the irregular network structure cannot maintain its structural integrity. This causes the shorter chain segments to fracture initially, which results in the development of microscopic cracks. The formation of these microcracks promotes stress concentration, eventually causing the emergence of macroscopic fractures.

**Figure 1:**
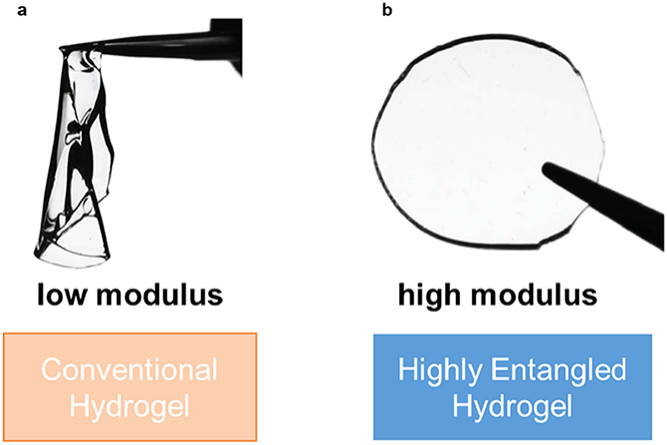
Schematic comparison of the two hydrogels. (a) Conventional hydrogel. (b) Highly entangled hydrogel. Reproduced from ref 37. with permission. Copyright 2021 American Association for the Advancement of Science.

To address this, researchers proposed various energy dissipation mechanisms to enhance and relieve the mechanical fragility of hydrogels.^5–8^ For instance, double-network hydrogels enhance the material’s strength and toughness significantly by constructing two interconnected polymer networks.^9–11^ Furthermore, nanocomposite hydrogels enhance the uniformity of the network structure and improve mechanical properties by incorporating nanomaterials;^12,13^ sliding-ring hydrogels dissipate energy through sliding crosslinking, which improves the material’s toughness and fatigue resistance;^14–16^ topological hydrogels improve energy dissipation mechanisms via the use of complex molecular chain design.^17–21^ Nevertheless, hydrogel materials face two major challenges at present:

The first challenge is the difficulty in balancing stiffness and toughness.^22^ Conventional hydrogels typically exhibit high water content and good flexibility, but these characteristics often result in inadequate rigidity, making them prone to plastic deformation or fracture. Conversely, enhancing the crosslinking density to improve the rigidity of the hydrogel leads to increased brittleness, decreased toughness, and a reduced capacity to endure large deformations.^23^

The second challenge concerns the balance between the polymer content and the material’s mechanical properties. On one hand, increasing the polymer content can significantly enhance the strength and modulus of the hydrogel, as a higher polymer concentration leads to a denser network structure and more physical or chemical crosslinking points.^24^ However, excessively high polymer content can cause the hydrogel to become more brittle, reducing its toughness and making it difficult to maintain integrity under large deformations.^25^

Some researchers introduced the strategy of entanglement to improve the mechanical performance of hydrogels. This strategy centers around improving the mechanical properties of the material by adding entanglement points between polymer chains, while avoiding excessive crosslinking that could make the material brittle (Figure 1b). As an effective method, this strategy has increasingly attracted the focus of many researchers within the polymer field.^26–28^

Entanglement results from the topological constraints caused by the interpenetration and entanglement of molecular chains, and it fundamentally originates from the geometric characteristics of multiple chain stacking due to the inaccessibility of the main chain.^29–32^ This phenomenon directly influences the rheological properties, crystallization dynamics, and mechanical behavior of polymers.^33^ Experimental studies have shown that the effective degree of entanglement (and its impact on rheological properties) is regulated by temperature, concentration, and shear forces.^34^

In this review, entanglement refers to topological constraints arising from the non-crossability of long polymer chains, without requiring specific bonding interactions. This differs from (i) chemical crosslinks (permanent covalent junctions) and (ii) physical crosslinks (energy-driven associations such as hydrogen bonding, ionic complexes, crystalline domains, hydrophobic aggregates, or nanoparticle-mediated junctions). In swollen hydrogels, the mechanical contribution of entanglement is inherently time-scale dependent: constraints formed in the preparation state may be partially retained as trapped entanglements after swelling, while other constraints can relax on longer time scales. We therefore discuss entanglement-controlled mechanics together with the relevant observation time and swelling history.^29–32^

To contrast entanglement-rich hydrogels with near-ideal polymer networks, we note that tetra-PEG gels are engineered via end-linking of well-defined tetra-arm precursors to minimize network defects and reduce entanglement effects, thereby providing benchmark systems for testing network theories.^35,36^ In contrast, this review focuses on hydrogels in which dense chain overlap and topological constraints are deliberately promoted (often by high-concentration polymerization or processing in a crowded state) to achieve robust mechanics under swelling. These two directions are complementary: near-ideal gels clarify baseline network physics, while entanglement-rich gels provide a practical route to overcome mechanical limitations of highly swollen hydrogels.

To facilitate a systematic understanding, this review is organized as illustrated in Figure 2. We begin by establishing the theoretical foundations and characterization techniques, followed by an analysis of critical factors and preparation strategies. Finally, we discuss the mechanical performance and diverse applications of entangled hydrogels.

**Figure 2:**
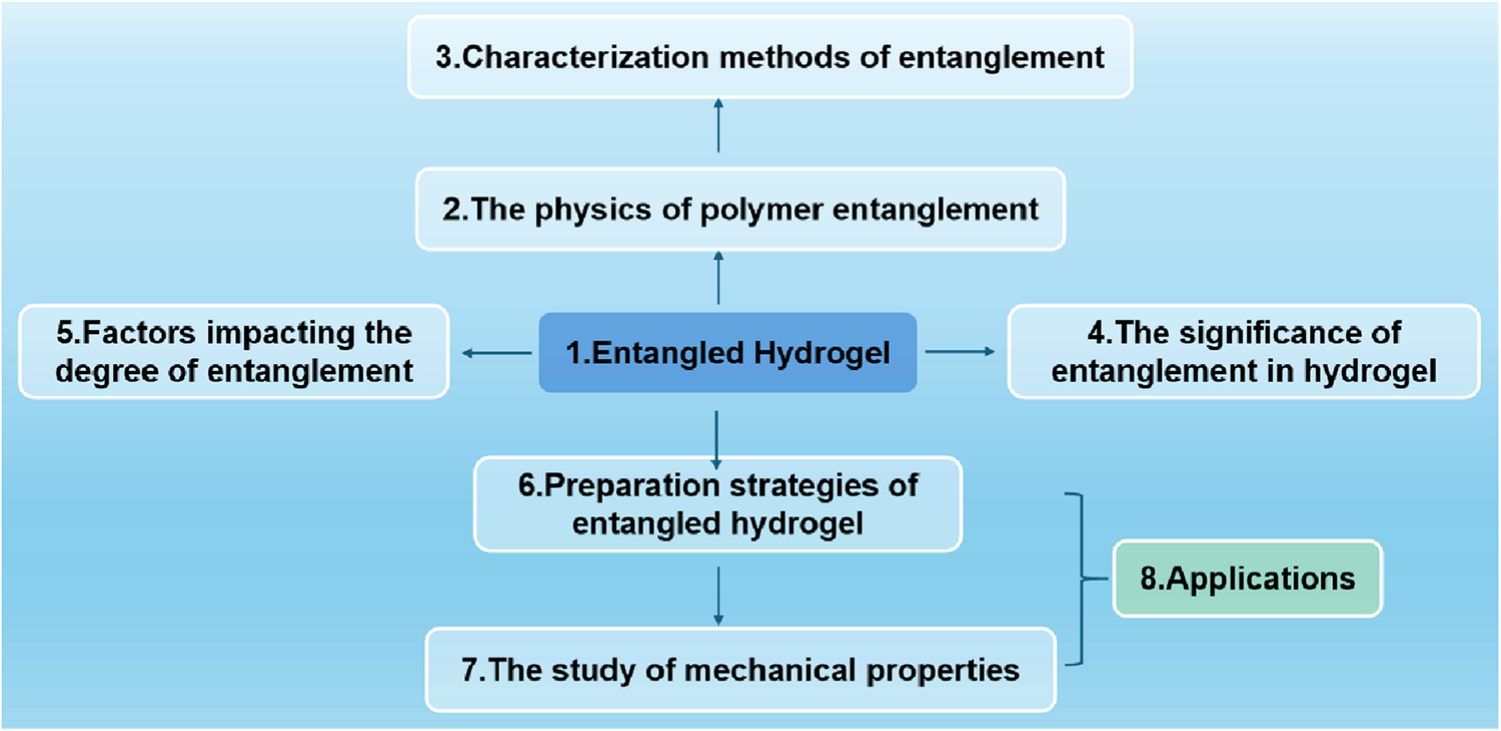
Roadmap and chapter association.

## 2 The Physics of Polymer Entanglement

Although experimental studies have widely confirmed the critical role of entanglements in hydrogels, such as enhancing mechanical properties and enabling rapid self-healing,^38–42^ the physical mechanisms underlying these phenomena still warrant in-depth investigation. Entanglements are not merely simple intertwinings of polymer chains, but topological constraints with structural functions, whose formation, evolution, and mechanical responses involve complex statistical physics and network dynamics.^43,44^

### 2.1 Topological Constraint and Tube Theory

The physical origin of entanglement is the fact that the covalent backbones of polymer chains cannot pass through one another (i.e., they are topologically inaccessible).^45^ In dense melts, the chains interlace with one another, forming complicated loops and knots. The configuration, determined by the relative positions of the chains, is a topological invariant that remains unchanged unless chemical bonds are broken. On the time scale required for polymer chains to fully disentangle, this topological structure remains preserved, greatly restricting the freedom of movement of each individual chain. For instance, a chain cannot simply move laterally to pass through another; it must instead resort to more complex and slower mechanisms to escape its surrounding constraints. It is precisely this challenging issue that led to the emergence of one of the most successful theories in polymer dynamics-the tube model (Figure 3).^43,46–48^ The tube model simplifies the complex multi-chain interaction acting on the test chain.

**Figure 3:**
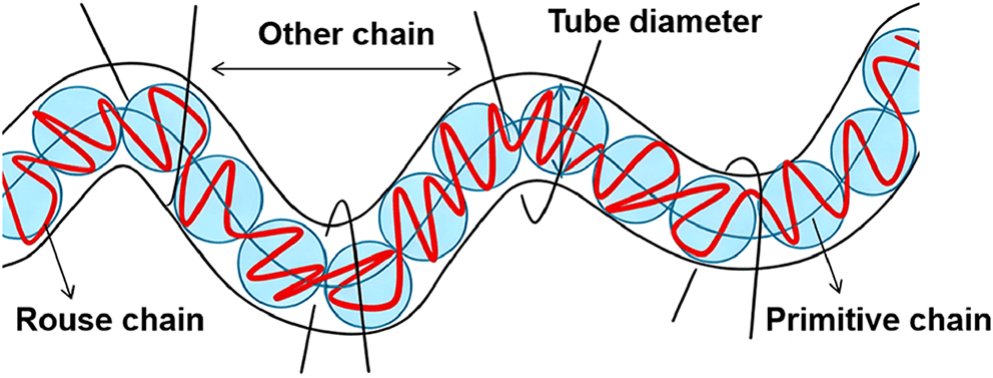
Schematic of the tube model.

To address the complex problem caused by topological constraints, de Gennes and Edwards proposed a revolutionary theoretical framework.^49^ The framework simplifies the problem to the motion of a single chain in a confined environment, thereby offering an intuitive and powerful physical model for understanding the dynamics of entangled polymers. The core idea of the tube model is to average the complex and discrete topological constraints exerted on a test chain by all other surrounding chains. These constraints are then averaged into a continuous, tubular potential field, known as the tube.^44,50,51^

After establishing the image of the tube as a static constraint, de Gennes further proposed a dynamical theory for how the chain moves under this constraint. de Gennes proposed that the primary macroscopic motion of a long chain confined within a tube is a “snake-like” or “worm-like” movement along its original path, which he termed reptation.^52–54^ This motion is driven by thermal fluctuations at both ends of the chain: one end randomly explores new space, forming a new segment of the tube, while the other end retracts from the old segment. The movement of the chain along the tube’s contour (primitive path) is modeled as a one-dimensional, curvilinear diffusion process.^55^

Classical tube/reptation ideas were developed mainly for polymer melts and concentrated solutions, where chain can constraints can renew or relax.^43,46,47^ In chemically crosslinked hydrogels, long-range chain diffusion is strongly hindered because network junctions effectively pin chain ends.^47,50^ Consequently, the topological constraints relevant to hydrogel mechanics are often trapped entanglements formed in the preparation state and partially retained after swelling, or time-dependent constraints whose contribution depends on the experimental time scale. Moreover, swelling decreases polymer volume fraction and thus reduces the effective entanglement density. Therefore, the tube model is used here primarily as a physically intuitive framework to describe topological confinement, while quantitative modeling of highly swollen networks must explicitly incorporate swelling, fixed junctions, and possible nonequilibrium history effects.

### 2.2 Entanglement Molecular Weight

Entanglement molecular weight is the molecular weight at which polymer chains start to exhibit significant entanglement. Once the polymer’s molecular weight surpasses this threshold, entanglement significantly impacts the rheological and mechanical properties of the material.^56,57^ The viscosity of the polymer solution or melt increases significantly, and the relationship between zero-shear viscosity and molecular weight follows a power-law relationship. In a polymer network, entanglement points can be considered as physical crosslinking, which enhance the material’s strength and modulus. The entanglement molecular weight determines the strength of interactions between chain segments, thereby influencing the material’s mechanical properties. Entanglements also significantly affect the crystallization process of polymers. Studies have shown that as the molecular weight increases, the degree of entanglement between polymer chains increases, resulting in a lower crystallization temperature.^58^

### 2.3 The Thermodynamics and Dynamics of Entanglement

#### 2.3.1 Thermodynamic Perspective

The entanglement of polymer chains is not a static, chemically crosslinked structure, but rather a dynamic phenomenon governed by physical principles. Essentially, entanglement formation is not driven by a specific attractive force or bonding energy, but is an unavoidable result dominated by entropy in dense systems.^58,59^ The core of this phenomenon is the interplay between the topological inaccessibility of polymer chains and the system’s tendency to maximize conformational entropy.

From a thermodynamic perspective, an isolated flexible polymer chain tends to coil into a random coil, as this configuration corresponds to the maximum number of conformational possibilities, thereby maximizing conformational entropy.^47^ However, in a polymer melt or concentrated solution, each chain attempts to maximize its own conformational entropy, while simultaneously being constrained by the surrounding thousands of other chains, each also striving for maximum entropy. The fundamental physical rule that polymer chains cannot penetrate each other forms the core topological constraint.^60^ To accommodate all the chains and allow them to stretch as much as possible, the chains inevitably interlace, forming loops and knots. These knots are the physical entanglement points. While this state limits the freedom of each individual chain, it is the inevitable outcome for the entire multi-chain system to achieve thermodynamic and kinetic equilibrium at a given density. The formation of the entangled network can be understood from the perspective of free energy.^59^ The Helmholtz free energy (*F* = *U* – *TS*) of the system must reach its minimum value. Here, the change in internal energy (Δ*U*) is minimal, so the system tends to maximize the total entropy (*S*).

The entangled state represents a balance: on one hand, the repulsive volume effects between chain segments are satisfied; on the other hand, the system’s overall conformational entropy is maximized, while maintaining the chains’ inaccessibility. Entanglement can be viewed as the topological cost the system pays to maintain high density and high conformational entropy. The entropy loss associated with this constrained state directly manifests in the material’s macroscopic elasticity. For example, when stretching a polymer material in an entangled state, its restoring force is essentially entropic elasticity, as the system attempts to return to a disordered state with higher conformational entropy.^61,62^ Researchers have proposed the concept of “topological entropy” to quantify the degree of entanglement.^58^ By calculating the number of different topological states (such as different types of knots) that can be formed in a system, a quantity directly related to entropy can be obtained. Studies showed that topological entropy is proportional to the classical entanglement length, providing deeper insights into the thermodynamic origins of entanglement.^58^

Notably, the emergence of entanglement-like topological constraints is not limited to highly flexible Gaussian coils. In concentrated solutions of semiflexible (or partly rigid) chains, neighboring polymers can confine a test chain into a tube primarily by restricting its transverse bending fluctuations, leading to an entangled regime with scaling laws distinct from flexible melts.^43,46,47^ Therefore, while reduced conformational freedom generally raises the concentration and/or molecular-weight requirements for entering an entangled regime, rigid or semiflexible polymers can still form long-lived topological constraints when the contour length is sufficiently large and the solution is sufficiently crowded.^63,64^ In polyelectrolytes, electrostatic stiffening and changes in effective chain diameter can further modify the entanglement spacing; additionally, specific physical associations (e.g., aromatic or ionic interactions) may coexist with entanglement and contribute to the apparent network stability.^65,66^

#### 2.3.2 Dynamic Perspective

Entanglement formation is a dynamical crossover-from fast, independent chain motion to slow, cooperative dynamics-not a thermodynamic phase transition with a well-defined critical point.^67,68^

For low molecular weight polymers, the chains are short and can move without severely hindering each other. This motion is described by the Rouse model,^69^ characterized by viscosity being proportional to molecular weight, and diffusion coefficient inversely proportional to molecular weight.^60^ When the molecular weight exceeds the critical threshold, the chain length becomes sufficient to form stable topological constraints. At this point, the chain’s motion is significantly restricted and must occur via slower mechanisms to escape the constraints. This leads to a sharp transition in the dynamic behavior, the reptation model predicts a much stronger dependence of viscosity on molecular weight.^70,71^

##### 2.3.2.1 Spontaneous Entanglement Process

The formation of entanglements is a multi-step process.^72^ Initially, simple topological structures, such as twists between chains or low-order knots, form rapidly. Subsequently, more complex and stable higher-order entanglements (such as higher-order knots or ring-like structures) take a longer time to develop.^73^

##### 2.3.2.2 Re-Entanglement after Flow

During strong shear or extensional flow, polymer chains are stretched and oriented, leading to partial disentanglement.^32^ Once the flow stops, the chains return to their random coil conformations through thermal motion, and the entangled network is restored.^74^ Molecular dynamics simulations and theoretical studies confirm that the re-entanglement process occurs at a very fast rate, with its characteristic time scale being the chain retraction time or Rouse time.^75^ This implies that once external stress is removed, the local topological constraint network can rapidly re-establish itself.

### 2.4 Modeling Approaches

To describe the complex dynamic behavior caused by topological constraints in polymer melts, physicists developed several theoretical models. The core objective of these models is to simplify a challenging many-body problem (the interaction of thousands of chains) into a computable and understandable physical image. These approaches vary in their level of abstraction and descriptive focus but collectively form the foundation of our understanding of the entanglement phenomenon. They can mainly be divided into: the tube model, which averages the constraints into a continuous tubular path of the original trajectory, and the transient network theory, which approaches from the perspective of macroscopic elasticity.

#### 2.4.1 Primitive Path and Tube Model

In a dense chain network, if we focus on a single test chain, its lateral motion is greatly restricted due to the hindrance from surrounding chains. By fixing both ends of the chain and allowing it to freely contract without intersecting with other chains, it will eventually settle into the shortest path. This shortest path, representing the macroscopic contour of the chain, is defined as the primitive path.^76^ The primitive path serves as a coarse-grained representation, eliminating the local, fast thermal fluctuations of the chain and preserving only the framework that governs the overall topological structure. In computer simulations, primitive path analysis is a powerful technique that can directly identify the entire system’s primitive path network by performing energy minimization on atomic-level simulation snapshots.^76,77^

The primitive path defines the trajectory of chain movement, whereas the tube defines the surrounding free space. The tube can be understood as a virtual tubular region enveloping the primitive path, with its diameter representing the average distance the polymer chain can freely move in directions perpendicular to its primitive path. This diameter quantifies the size of the “cage” formed by surrounding chains. Therefore, the tube model averages the discrete, dynamic topological constraints into a continuous, static potential field, greatly simplifying the problem.^78,79^ The chain’s motion is confined within this tube, and the primary relaxation mechanism is reptation along the tube.

The core advantage of the primitive path and tube model is the intuitive and simplified physical image. It successfully transforms a many-body problem into a single-chain problem and provides powerful scaling law predictions for properties such as viscosity and diffusion coefficients.

#### 2.4.2 Transient Network Theory

The transient network theory offers a more macroscopic, thermodynamic framework for understanding entanglements. It treats the entangled melt or concentrated solution as a physical, temporary network structure, analogous to the chemically crosslinked rubber. In this theory, the topological entanglement points between chains are considered as temporary network nodes or physical crosslinking, with the segments connecting these nodes referred to as network chains.

When the material is deformed, if the time scale is shorter than the time required for the chains to untangle, this transient network responds like a permanently crosslinked rubber, exhibiting elasticity.^41^ This elasticity is fundamentally entropic in nature: deformation stretches the network segments from the most probable random coil conformation, leading to a decrease in system entropy. Upon removal of the external force, the system spontaneously returns to a high-entropy state, generating a restoring force. This rubber-like behavior is observed in stress relaxation experiments as a flat plateau region, and the modulus value is referred to as the plateau modulus.

The core contribution of the transient network theory is that it directly relates the measurable plateau modulus to the microscopic entanglement density. The plateau modulus is proportional to the number of network chains per unit volume, which can be expressed using the entanglement molecular weight (*M*_e_).

Indeed, according to the Edwards tube model, the modulus of the entangled polymer network can be approximated as shown in equation.^80^

$$G=G_{x}+G_{e}\approx \rho \mathrm{RT}\left(\frac{1}{M_{x}}+\frac{1}{M_{e}}\right)$$

Here, *G*_*x*_ and *G_e_* represent the contributions of crosslinking points and entanglements to the modulus, respectively; *M_x_* is the average molecular weight between crosslinking points, *M_e_* is the average molecular weight between entanglements, and ρ is the polymer density. It can be seen that when *M*_*x*_ is sufficiently large, the contribution of the crosslinking should become negligible, the modulus is determined almost entirely by *M_e_*, indicating that the system formed a highly entangled hydrogel network.

This equation is one of the cornerstones of polymer physics, as it allows us to quantify the material’s microscopic entanglement density through macroscopic rheological measurements.^44^ These two modeling methods are not in conflict with one another; instead, they offer complementary perspectives that describe the same physical reality at different levels. The development trend in modern polymer dynamics theory is to integrate the advantages of these models.

## 3 Characterization Methods of Entanglement

The essence of entanglement is not discrete chemical crosslinking but rather a topological constraint network formed by polymer chains interlocking and restricting each other’s movement. The characteristics of this network cannot be fully captured by any single technique. Therefore, a comprehensive characterization framework should first approach from the macroscopic level, quantifying the contribution of entanglements to key material properties such as overall stiffness, toughness, energy dissipation (hysteresis), and viscoelasticity through fundamental mechanical testing. At the nanoscale, scattering techniques, particularly Small-Angle X-ray Scattering (SAXS) and Small-Angle Neutron Scattering (SANS), are required to obtain statistical structural information about network correlation lengths, mesh size distributions, and chain segment aggregation states. To explore the physical origins of entanglement phenomena, molecular-level investigations must be conducted, employing techniques like Dielectric Relaxation Spectroscopy (DRS) and Nuclear Magnetic Resonance (NMR) spectroscopy. These spectroscopic methods can directly probe the dynamic behavior of polymer chain segments over different time scales, such as global relaxation and local motion, revealing the limiting effects of topological entanglements on chain movements.

### 3.1 Mechanical Properties Characterizations

#### 3.1.1 Uniaxial Testing: Stress-Strain Curve

A typical stress-strain curve reveals a series of behavioral stages during the stretching process of the material, reflecting its mechanical properties.^81,82^ Toughness is a comprehensive measure of a material’s resistance to fracture, defined as the total energy absorbed by the material from the initiation of stress to the point of fracture. Numerically, it is equal to the total area under the stress-strain curve.^83,84^ Toughness combines a material’s strength and ductility; a material with high toughness must be both strong and resistant to breaking.

Microscopically, strain hardening in hydrogels can originate from multiple mechanisms. One contribution is non-Gaussian chain elasticity (finite extensibility), where network strands progressively approach their maximum stretch.^85,86^ In addition, in entanglement-rich networks, topological constraints can be described as sliplink- or tube-like sliding junctions at moderate strain: chains can redistribute contour length along the backbone, which helps avoid concentrating stress in the shortest strands. At sufficiently large deformation, however, contour-length redistribution becomes increasingly restricted as chains align and the effective tube tightens; the system enters a locking regime in which entanglement constraints behave more like fixed junctions, producing a pronounced increase of the tangent modulus.^50,56^ In this view, increasing the effective entanglement density (or decreasing the effective strand length between constraints) typically shifts the onset of locking to lower strain and strengthens the hardening response.

#### 3.1.2 Cyclic Loading and Energy Dissipation Mechanism

Cyclic loading-unloading testing is a crucial method for investigating a material’s energy dissipation and recovery capabilities.^87^ During the test, the sample is stretched to a predetermined strain and then unloaded to zero stress, with the entire stress-strain path recorded. For an ideal purely elastic material, the loading and unloading curves will overlap completely. However, for viscoelastic materials like hydrogels, the unloading curve always lies below the loading curve, with the two forming a closed loop known as the hysteresis loop. The area enclosed by this loop physically represents the energy dissipated per unit volume of material due to internal friction and other inelastic processes during one loading-unloading cycle.

The fatigue resistance of a material is closely related to its hysteresis behavior. Materials that dissipate energy through structural damage (such as the breaking of sacrificial bonds) exhibit high hysteresis, and their damage accumulates over cycles, resulting in typically poor fatigue resistance.^4^ In contrast, materials with reversible, low-dissipation mechanisms (low hysteresis) exhibit excellent fatigue resistance, as their network can largely recover after each cycle.^88–90^ Therefore, the shape and size of the hysteresis loop serve as powerful tools for diagnosing the internal energy dissipation mechanisms of materials. A wide, open loop suggests that the material’s toughness primarily arises from irreversible energy dissipation processes such as sacrificial bond breaking. In contrast, a narrow, closed loop implies that the material’s toughness is derived from more recoverable, entropy-elastic network rearrangement mechanisms. This distinction is crucial for material design: the former is suitable for one-time energy dissipation scenarios such as impact absorption, while the latter is ideal for elastic components that require long-term, stable load-bearing. The development of novel materials like double-network entangled hydrogels,^9–11,91^ exploit the toughening mechanism of physical entanglements without sacrificial bond.

### 3.2 Rheological Characterization of Entanglement

Rheology is the science of studying material deformation and flow. By applying controlled stress or strain and measuring the response, it explores the internal structure and dynamics of materials for polymer melts and concentrated solutions, rheological behavior provides the most direct and sensitive macroscopic representation of the entangled state. Therefore, rheological measurements, particularly linear viscoelastic measurements, have become the most classical and widely used method for quantitatively characterizing entanglements. Akcora et al. used rheological methods to characterize the composite network system of poly­(methyl acrylate) with entangled and non-entangled structures.^92^

Small-amplitude oscillatory shear is a core experimental technique for characterizing the linear viscoelasticity of polymers.^93,94^ By changing the oscillation frequency (ω), we can explore polymer dynamics across different time scales. For high molecular weight entangled polymer melts, the curves of the storage modulus (*G*′) and loss modulus (*G*″) as a function of frequency exhibit distinct characteristics that clearly reveal the presence of entanglements.

### 3.3 Scattering and Microscopy in Entanglement Studies

During deformation of soft materials, the dynamical behavior of polymer chains is constrained by entanglements, which can be observed using techniques such as SANS, SAXS, and polarized optical microscope (POM). SANS and SAXS are employed to detect changes in chain density anisotropy at the nanoscale caused by entanglements during deformation, with these changes becoming evident through alterations in scattering patterns induced by strain. In the deformation process, the entangled structure slips under stress, causing polymer chains to align along the direction of the applied force, which increases birefringence. The POM technique can then be used to observe the orientation of polymer chain segments through birefringence.

Since crystalline regions generally produce strong scattering signals, Snyder et al. utilized SANS to measure the molecular volume fraction in the amorphous regions of deuterated solvent-swollen materials.^95^ The results indicated a correlation between the volume fraction and the number of entanglement points. Olsen et al. used SAXS and in-situ POM techniques to observe the structural changes of hydrogel under uniaxial strain, with engineering strains as high as 2000%.^96^ The results revealed that the entangled hydrogel exhibited anisotropy and significant non-monotonic birefringence during deformation, confirming that molecular entanglements promote the extensive stretching and alignment of macromolecular chains, thereby effectively bearing stress. Wang et al. used POM to observe highly entangled hydrogel samples, both with and without notches, under varying tensile strains.^91^ The birefringence index was measured in the central region of the hydrogel and calculated using a formula. Additionally, a custom-made manual stretching device was used to simultaneously record the in-situ stretching dynamics of the samples with and without notches.

The anisotropic optical response to uniaxial strain across nano, micro, and macroscopic scales revealed that the stress dissipation mechanism is crucial for achieving high extensibility and toughness.

### 3.4 Dielectric and Nuclear Magnetic Resonance (NMR) Spectroscopy

While rheology infers entanglement from macroscopic response, and scattering and microscopy attempt to observe entanglement from static structures, spectroscopic methods take a different approach by probing molecular motion at different time scales to detect the dynamic constraints imposed by entanglements. When chain movement is restricted by entanglements, characteristic changes appear in the spectral signals. Dielectric spectroscopy is an effective method for measuring the frequency-dependent variation of a material’s dielectric properties, such as dielectric constant and dielectric loss, under an alternating electric field.^97^ Drakopoulos’ research team employed dielectric spectroscopy, a technique that measures the dependence of the complex dielectric constant on electromagnetic frequency,^98^ to study the entanglement structure in ultrahigh molecular weight polyethylene.^99^ To achieve this, polyethylene was lightly oxidized to introduce a small amount of “dielectrically active” functional groups. By comparing the dielectric data with the mechanical shear modulus, they found that in highly entangled regions, these dielectrically active groups exhibited a slow response to electric field changes and reduced sensitivity to high-frequency electromagnetic waves. Based on this, the Drakopoulos team identified characteristic frequencies of the disentangled and entangled amorphous polymer phases. A similar concept was demonstrated in the work of Sprakel and Guo’s research teams, who used X-ray photon correlation spectroscopy to observe how nanoparticle diffusion is influenced by molecular entanglement.^100,101^

The use of multi-quantum nuclear magnetic resonance (MQNMR) also allows for the observation of the “slow motion” of molecular chains in entangled regions.^102^ When topological constraints are present, the movement of polymer chains in hydrogels and elastomers exhibits anisotropy,^103^ leading to a non-zero average dipolar coupling between protons, known as residual dipolar coupling. This coupling signal would relax over time and is highly sensitive to the polymer’s degrees of freedom, making it an effective probe for studying trapped entanglements.^104^

In a study on semicrystalline polymers, the Jin team used NMR and fitting methods to investigate how entanglements in the amorphous region affect the interconnection of crystalline domains.^105^

## 4 The Significance of Entanglement in Hydrogel

The conflict between stiffness and toughness in hydrogels, as well as the imbalance between polymer content and mechanical performance, has been widely recognized, prompting numerous polymer scientists to focus their efforts on addressing these two critical challenges. Thus, the concept of entanglement has garnered increasing attention among researchers. Entanglement in hydrogels can markedly enhance their mechanical properties by reinforcing stress transfer, providing energy dissipation pathways, and stabilizing the network structure. Compared to conventional hydrogels, entangled hydrogels exhibit significant advantages in stiffness, toughness, strength, fatigue resistance, and frictional behavior.^37^

### 4.1 Overcoming the Conflict between Stiffness and Toughness

From a mechanical perspective, entanglements serve as sliding linkages. When the hydrogel is subjected to external stretching forces, the entangled polymer chains can slide and elongate. This sliding mechanism enables tension to be transferred along the chains and transmitted through entanglements to many other chains. Even if one polymer chain ruptures, its stress is transferred through entanglements to many other chains. Because entangled chains effectively share loads, the break of a single bond does not cause the material to fail. This load redistribution mechanism delays fracture and thus substantially enhances toughness.^37,91,106^ Compared with conventional crosslinking approaches, entanglements do not cause the polymer to become brittle. When the number of entanglements greatly exceeds that of chemical crosslinking, they can act as additional crosslinking points, increasing the stiffness of the polymer while simultaneously enhancing toughness.

Here it is helpful to distinguish sliding from stress delocalization. Sliding (or contour-length redistribution) describes the local kinematics enabled by topological constraints, whereas stress delocalization refers to the resulting redistribution of tensile load over a longer contour length and a larger effective process zone. By transmitting force away from the most highly stressed local strands, delocalization mitigates stress concentration and delays catastrophic crack growth. Recent designs based on unusually long polymer chains crosslinked by domains of physical bonds provide a clear illustration of this concept: long chains can engage multiple domains so that crack-tip tension is transmitted over extended segments rather than being localized to a short strand, enabling high modulus together with high fatigue resistance.^107,108^

On the microscopic level, conventional hydrogels are generally composed of a net-like network structure, where crosslinking predominates over entanglement. In contrast, highly entangled hydrogel polymers exhibit a fabric-like topology where entanglements exceed crosslinking. This configuration allows for extensive interchain weaving and structural reinforcement, resulting in enhanced stress distribution and diminished stress concentration.

In these polymers, the segments between crosslinking points are remarkably long, allowing entanglements to function as highly slidable junctions. When highly entangled polymers are stretched, they form an orderly, fabric-like topology. Before any individual polymer chain breaks, the stress is effectively transferred through dense entanglements to surrounding chains (Figure 4). Consequently, when a single covalent bond breaks, the polymer dissipates not only the elastic energy of the fractured chain but also that of multiple interconnected chains, ultimately contributing to elevated toughness. Hence, high stiffness and high toughness are not mutually exclusive in such entangled systems.

**Figure 4:**
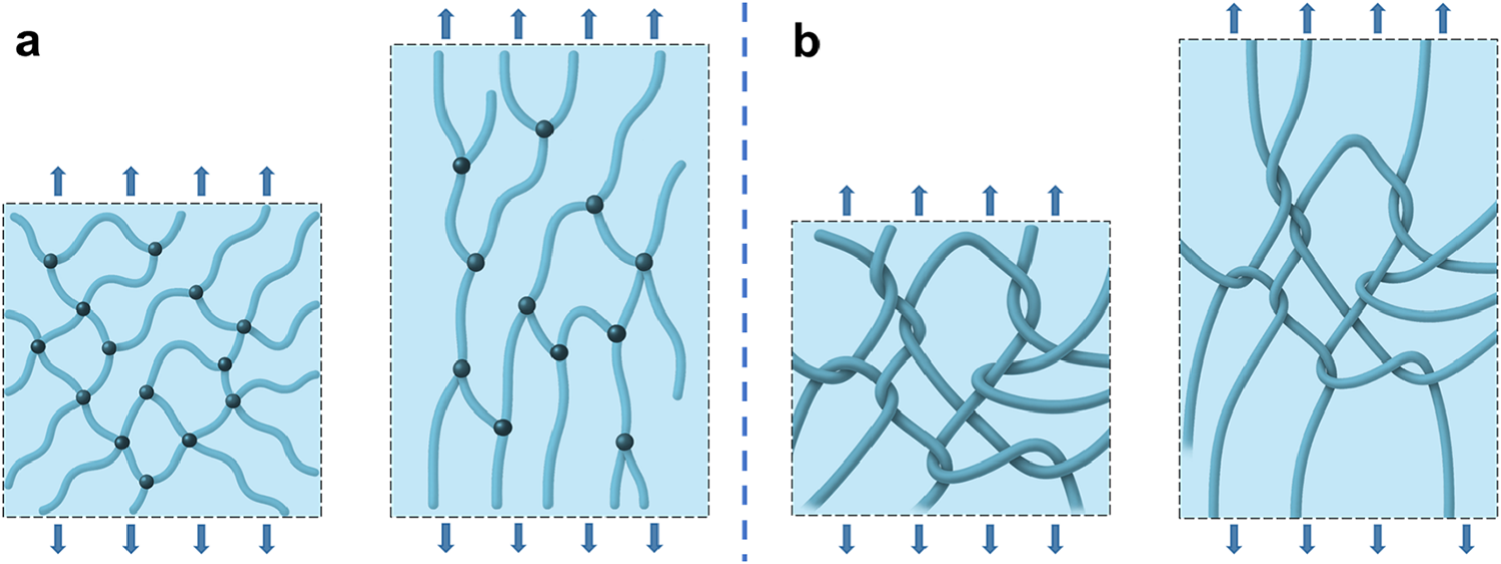
Schematic of conventional and highly entangled hydrogels when stretched. (a) Conventional hydrogel. (b) Highly entangled hydrogel.

### 4.2 Overcoming the Imbalance between Polymer Content and Mechanical Properties

As the polymer content increases, the strength and modulus of hydrogels typically improve due to the formation of denser network structures, which enhance the material’s resistance to deformation. Within a certain range, increasing polymer content can enhance the toughness of hydrogels, but surpassing a critical threshold may lead to embrittlement and reduced mechanical resilience. In contrast, highly entangled hydrogels enhance toughness by strengthening interactions among polymer chains, without the brittleness typically associated with high crosslinking densities.^109^

### 4.3 Other Advantages

In terms of mechanical properties, the entanglement of polymer chains significantly reinforces the material’s strength and toughness. Regarding rheological properties, entanglements in the precursor solution elevate its viscosity,^110^ in the final cured hydrogel, they contribute to a higher elastic modulus. For solubility, highly entangled polymers exhibit reduced solubility due to the need to overcome extensive internal entanglements during dissolution.^111^ In diffusion and permeability, polymer entanglements restrict the diffusion of small molecules or particles, thereby modulating the material’s permeability.^112^ Concerning aging and creep behavior, under prolonged loading, polymers typically undergo creep. However, entanglements can slow down the creep rate, enhancing the long-term stability of the material.^113^ In terms of self-healing, the entanglement and subsequent disentanglement of polymer chains form the fundamental mechanism underlying self-healing behavior.^114,115^ In molecular motion, entanglements limit the segmental mobility of polymer chains, thereby influencing the material’s movement dynamics and thermodynamic properties.^116,117^ As for the friction coefficient, as hydrophilic polymers, hydrogels stabilize a hydration layer via their polymer chains, leading to surface lubrication. The thickness of this lubricating layer increases with chain length, resulting in a lower coefficient of friction.^118,119^ Regarding fatigue resistance, Under cyclic loading, the redistribution of tension through entanglements elevates the fatigue threshold, addressing the issue of high hysteresis caused by the slow recovery of sacrificial bonds.^118^ Anti-swelling properties of entangled hydrogels stem from their network architecture: a high density of entanglements can physically constrain the polymer network from expanding excessively in water, even when chemical crosslinking density is low. In a highly entangled network, polymer chains are so topologically interwoven that the hydrogel maintains structural integrity upon swelling, preventing it from dilating or dissolving.^120^ These characteristics enable highly entangled hydrogels to maintain structural integrity and consistent performance during swelling, making them well-suited for applications demanding high stability and durability. In terms of biocompatibility, entanglements serve as robust physical crosslinking that enhance the hydrogel’s compatibility with biological systems.^121–123^ The network of highly entangled hydrogels is primarily constructed through polymer entanglement, which substantially lowers the reliance on chemical crosslinking, thereby reducing irritation and toxicity to surrounding tissues during application. In addition, entangled hydrogels can be synthesized using naturally derived polymers like hyaluronic acid and gelatin, which significantly improve their biological compatibility. Their inherent high water content and porous morphology make them well-suited for a broad range of applications in the biomedical field.

As a new generation of soft materials, entangled hydrogels overcome the mechanical limitations of conventional hydrogels through biomimetic network topology design. Their distinctive dynamic physical entanglement mechanism and energy dissipation pathways endow them with exceptional stretchability, toughness, and rapid self-recovery. These properties offer innovative solutions to pressing issues in biomedicine, flexible electronics, and energy, thereby advancing scientific progress and innovation.

## 5 Factors Impacting the Degree of Entanglement

Entanglement, as a unique physical phenomenon in polymer materials, plays a crucial role in determining various material properties. This topological constraint, formed by the interpenetration and entanglement of polymer chains, is not a result of chemical crosslinking, yet it functions similarly to physical crosslinking points. Understanding and controlling the degree of entanglement is crucial for the design, modification, and application of polymer materials.

### 5.1 Impact of Polymer Concentration

In solution or blend systems, the concentration of polymers directly determines the distance between chains, thereby influencing the formation of entanglements. Beyond concentration, solvent quality and polymer–solvent affinity can strongly modulate entanglement by changing chain dimensions and overlap conditions.^47,57^ In a better solvent, chains are more expanded and can reach the overlap/entangled regime at lower mass fraction, whereas poorer-solvent conditions can contract coils and may require higher concentration to achieve comparable interpenetration.^47^ For polyelectrolytes, ionic strength and counterion screening further alter effective stiffness and chain–chain interactions, thereby shifting the entanglement spacing.^65^ Consequently, when discussing high entanglement processing routes (especially in solvent-rich systems), it is important to specify not only polymer content but also solvent conditions and polymer hydrophilicity that control chain conformation during network formation and subsequent swelling.

In the dilute region, where concentration is below the critical entanglement concentration, polymer chains exist as discrete coils without overlap, leading to a near absence of entanglement.^124^ In the semidilute and concentrated regions, rising concentration leads to increasing contact, overlap, and entanglement among polymer chains. Once the concentration exceeds the critical entanglement threshold, interchain entanglements become prominent and intensify with further concentration increase, leading to the formation of a continuous entangled network and a sharp rise in solution viscosity. In the case of a specific polymer, the higher the molecular weight, the lower the value of the critical entanglement concentration, implying that higher molecular weight polymers are capable of forming efficient entanglements at reduced concentrations.^125^

### 5.2 Internal Regulation of Polymer Chain Flexibility

The inherent chemical structure of a polymer chain dictates its flexibility, which directly influences its ability to adopt coiled conformations in space and, consequently, its propensity to form entanglements.^126^

Flexible polymer segments exhibit greater mobility and curvature, facilitating interpenetration and the formation of entanglements. Consequently, flexible-chain polymers typically exhibit lower entanglement molecular weights and higher entanglement densities. When the backbone of a stiff polymer chain contains bulky groups such as benzene rings, heterocycles, or conjugated structures, internal rotation is hindered, leading to reduced chain flexibility. This hinders close packing and effective interpenetration between chains, resulting in a reduced entanglement density and typically a higher entanglement molecular weight. For instance, polystyrene exhibits a higher entanglement molecular weight than polyethylene, owing to the presence of bulky phenyl side groups.

### 5.3 Impact of Temperature on Entanglement Dynamics

Temperature significantly influences the dynamic behavior of entangled networks by affecting the mobility of polymer chains.

As the temperature rises, the thermal motion of polymer chain segments intensifies, providing them more energy and free volume. This enables the chains to undergo disentanglement and re-entanglement processes more rapidly, accelerating the formation and disappearance of entanglement points.^127^ Macroscopically, the material exhibits reduced viscosity and enhanced flowability. During high-temperature processing, this effect allows the material to be molded and shaped. Below the glass transition temperature, large-scale segmental motion (such as reptation) effectively ceases and the entanglement configuration becomes fixed, causing the material to exhibit a stiff, glassy state. In the rubbery state above the glass transition temperature, the entanglement network imparts rubber-like elasticity to the material.

## 6 Preparation Strategies of Entangled Hydrogel

### 6.1 High Concentration Monomer

Using a high monomer concentration is a crucial method. Earlier, Zhigang Suo and colleagues proposed this method. As monomer concentration rises, polymer chains are more likely to interact and intertwine, resulting in a network structure enriched with physical entanglements that contribute to improved mechanical performance (Figure 5).^128^ Research by Suo et al. demonstrated that a highly entangled hydrogel can be fabricated by formulating a precursor solution with large numbers of monomers, minimal amounts of water, crosslinker, and initiator.^37^ The crowded monomer environment in the precursor solution induces a high degree of entanglement in the resulting polymers. The study confirmed entanglements by preparing single-network systems where the entanglement density greatly exceeded the crosslinking density (Polyacrylamide hydrogels, polyacrylic acid hydrogels, and acrylate elastomers).

**Figure 5:**
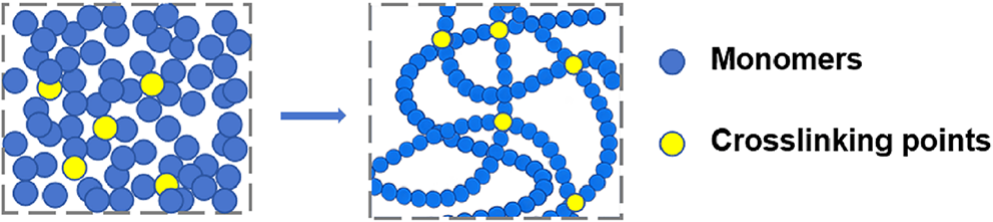
Schematic of high concentration monomer method.

Zhang et al. reported a robust and self-healing hydrogel with a modulus of 50,000 kPa and a tensile strength reaching 4200 kPa, achieved through polymer entanglement within coplanar nanoscale confinement.^40^ It was prepared by polymerizing a highly concentrated monomer solution within a scaffold of fully exfoliated synthetic lithium montmorillonite nanosheets, followed by shear alignment to generate a macroscopical single-domain structure. Despite its high modulus, the resulting physical gel exhibits a self-healing efficiency of up to 100% and demonstrates strong adhesive shear strength across a variety of substrates. This nanoconfinement strategy enables the incorporation of new functionalities by embedding colloidal materials and can be extended to other polymers and solvents to fabricate stiff and self-healing hydrogels for applications in soft robotics, additive manufacturing, and biomedicine.

### 6.2 Kneading and Annealing

Conventional hydrogels are formed by chemically crosslinking preformed polymer chains into a network structure, which enhances swelling resistance but compromises mechanical toughness. This conflict is resolved through a kneading-annealing approach to hydrogel fabrication, which is compatible with industrial processing techniques and paves the way for the development of sustainable, high-performance hydrogels (Figure 6).

**Figure 6:**
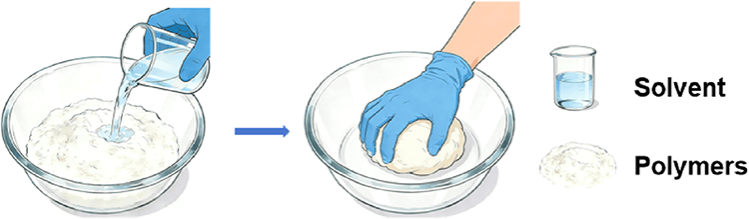
Schematic of kneading dough method.

Suo et al. formed a heterogeneous and opaque dough by mixing powdered polyethylene glycol polymer chains with a minimal amount of water and a photoinitiator.^129^ A kneading cycle involves folding the dough twice at 80 °C, compressing it back to its original thickness within 2 min using aluminum plates, and maintaining the pressure for 9 min. After seven kneading cycles, the dough becomes homogeneous and transparent, followed by overnight annealing at 65 °C. The combined kneading and annealing at elevated temperatures homogenize the dough, during which the densely packed polymer chains become tightly entangled. The material is then sparsely crosslinked into a polymer network under ultraviolet light and subsequently immersed in water to swell to equilibrium. The resulting hydrogel is not only resistant to swelling and mechanically robust, but also exhibits near-perfect elasticity, high strength, excellent fatigue resistance, and low friction.

Li et al. synthesized a polyelectrolyte complex hydrogel.^130^ The hydrogel was fabricated from a precursor dough by kneading, annealing, and crosslinking two oppositely charged polysaccharides—cationic quaternized chitosan and anionic sodium hyaluronate—along with a photoinitiator (β-ketoglutaric acid), a crosslinker (glycidyl methacrylate), and a minimal amount of water. Controlled kneading and annealing homogenize the dough, transforming randomly distributed individual polymer chains into tightly entangled double-chain structures. The tightly entangled network is then formed through sparse covalent crosslinking under UV irradiation, and after full swelling in water, yields the polyelectrolyte complex hydrogel. This hydrogel exhibits near-perfect elasticity, high tensile strength, and excellent resistance to swelling.

Inspired by dough kneading, Huang et al. rapidly mixed hydrophobic particles with a polyethylenimine polymer solution to form a dough-like substance, ensuring homogeneous dispersion of the hydrophobic particles within the polymer substrate, thereby facilitating the formation of a stable suspension in water solution.^131^ By integrating photopolymerization or thermal curing, a composite hydrogel incorporating hydrophobic particles into polyethylenimine-polyacrylamide was synthesized, featuring excellent self-healing capability and adjustable mechanical properties. Incorporating hydrophobic particles into the hydrogel network significantly reduced the swelling ratio and increased the compressive modulus by over five times.

### 6.3 Other Strategies

Apart from the two methods discussed, a wide range of other methods can also be employed to construct entangled hydrogels. For example, double-network hydrogels fabricated using ion-covalent entangled networks leverage multiscale entanglements formed by molecular entanglement and ionic cluster crosslinking, successfully integrating the stability of covalent bonds with the dynamic nature of ionic interactions to create entangled hydrogels featuring high strength, reversible self-healing, and environmental responsiveness;^132–135^ by allowing monomers or oligomers to polymerize in situ, and then physically entangle with nanomaterials. The efficiency of stress transfer within the hydrogel network is significantly enhanced;^136,137^ by combining different polymer chains through physical entanglement and interactions such as hydrogen bonding and coordination bonding, an enhanced network structure is achieved;^103,138,139^ by designing an asymmetric molecular entanglement node, a woven network that combines rigidity and flexibility has been created;^140^ by introducing multiphase components with distinct chemical or physical properties, topological entanglement networks can be formed through the intertwining of polymer chains, thereby achieving mechanical properties and functionalities that are difficult to realize in homogeneous hydrogels.^141^ These approaches either enhance entanglement through distinct mechanisms or optimize hydrogel functionality by introducing entangled structures, offering diverse strategies for the development of high-performance hydrogels. Sustainability is also important for hydrogel synthesis, for example, recent work highlights waste-to-hydrogel routes as a circular manufacturing approach.^142^

Beyond polymerization in a crowded state, solvent exchange can also strengthen and stabilize network constraints by changing polymer–solvent interactions after gelation. For example, a polymer gel formed in a strongly solvating medium (e.g., DMSO or related solvent environments) can be transformed into a hydrogel by solvent replacement, during which interchain hydrogen bonding, phase separation, or microstructural densification can emerge and help kinetically trap chain interpenetration. Such solvent-history effects can enhance swelling resistance and mechanical robustness even at high water content. Carbon dots/clusters and biomass-derived carbon nanomaterials have been reported to strengthen hydrogels by crack-arresting (“pinning”) effects and strong interfacial interactions with polymer chains.^143,144^ In addition to serving as reinforcement/physical junctions, these nanostructures can promote dense chain interpenetration during polymerization and thereby increase the effective constraint density that resists crack propagation.

## 7 The Study of Mechanical Properties

### 7.1 Single Entanglement Factor

Currently, the primary role of entanglement in hydrogels is to enhance mechanical performance. Table 1 shows a comparison of the mechanical properties of entangled hydrogels.

**Table 1:** Comparison of Mechanical Properties of Entangled Hydrogels

Component	Crosslinker	Elongation at break (%)	Toughness (kJ m^–3^)	Fracture toughness (kJ m^–2^)	Breaking strength (kPa)	Elastic modulus (kPa)	Other energy dissipation mechanisms	References
PAAm^a^	MBAA^b^	≈270	—	≈8	390	≈110	—	^37^
PAAm^a^, AMPS^c^	MBAA^b^	≈340	2490	8.34	3000	—	Double network	^85^
PAAm^a^	MBAA^b^, BACA^d^	—	—	≈1	—	≈100	—	^141^
Ferritin-like proteins(FL)	tris(bipyridine) ruthenium(II)	107 ± 14	250 ± 68	—	—	700 ± 110	—	^165^
PAAm^a^, ZrO_2_, LiBr	MBAA^b^	330	—	—	360	—	—	^166^
PAAm^a^, PMPC^e^	MBAA^b^	2000	1600	4	≈10,000	≈130	—	^167^
P(AAm-AAc)^f^	MBAA^b^	560	42,600	—	10,200	8300	Coordinate bond	^157^
P(AAm-AAc)^f^	MBAA^b^	920	—	—	470	—	—	^168^
PEG^g^	—	≈700	854	2	6500	≈800	—	^129^
HACC^h^, HA^i^	glycidyl methacrylate	238	530	—	474	≈3400	—	^129^
PAAm^a^	MBAA^b^	300 ± 20	260 ± 4	—	170 ± 30	83 ± 10	Double network	^131^
PEGDGE^j^;κ-Carrageenan, CaCl_2_, Jeffamine^k^	—	350 ± 60	1400 ± 300	—	600 ± 70	320 ± 40	Double network	^132^
gellan gum, Soy protein isolate	KCl, MTGase^l^	54 ± 2	—	—	8.1 ± 0.3	10.2 ± 1.3	Double network	^133^
Carrageenan, Gellan gum, Epoxy-amine	CaCl_2_	≥80	—	—	930 ± 130	120 ± 57	Double network	^134^
PVP^m^,PI^n^, DMF^o^	—	≈42	—	—	2140	22570	—	^169^
PEG^g^, PLA^p^	—	≈700	4000	≈11	≈700	4000	Semi-crystalline domain	^164^
PEGDA^q^, PEG^g^	—	2890 ± 2 70	912.5 ± 52.3	—	76.0 ± 11.2	—	—	^163^
PAAm^a^, Polyacrylamide nanogels	—	>600	—	—	—	1	—	^109^
NIPAm^r^, Hydroxypropyl cellulose	MBAA^b^	≈500	114 ± 0.2	—	≈50	20.0 ± 0.2	Hydrogen bond	^136^
PAAC^s^, Polyaniline	MBAA^b^	2830	1540	—	120	—	—	^170^
PAAM^a^, poly(acrylate sodium) microgels	MBAA^b^	≈430	—	—	990	112.45	—	^162^
PAAm^a^, AAC^t^	MBAA^b^	1229.02 ± 80.62	≈2600	—	630 ± 420	≈80	—	^161^
PEG^g^	α-Cyclodextrin	1500	—	—	1840	—	slide-ring	^171^
protein	—	1040 ± 210	210 ± 42	—	—	—	—	^96^
Gelatin, ZnSO_4_	Oxidized Dextran, Methacrylic anhydride	483.3	—	—	6800	—	Multi-network	^172^
HEAc^u^, Agarose	—	999	4510	—	820	—	—	^150^
PMMA^v^	—	570	—	—	120	—	—	^140^
Protein	—	3000	65	—	>300	15.30 ± 0.65	—	^173^
PAAm^a^, PAMPS^w^	MBAA^b^	—	—	1	20,000	300 ± 40	Double network	^174^
PAAm^a^, PDGI^x^	MBAA^b^	1180	4380	—	150	≈400	Hydrogen bond	^138^
PEA^y^, PAAC^s^	MBAA^b^	≈520	—	≈1.7	6900	443	Phase separation	^175^
PAAm^a^, PAMPS^w^	MBAA^b^	360	—	4202	≈250	40	Sequential polymerization	^150^
AAC^t^, PVA^z^, acrylamide, Chitosan	Ca^2+^	500	2580	—	1200	360	Controlled polymerization	^106^

a Polyacrylamide.

b *N*,*N*’-Methylene biacrylamide.

c 2-Acrylamido-2-methylpropanesulfonic acid.

d *N*,*N′*-Bis (acryloyl) cystine.

e Poly­(2-methacryloyloxyethyl phosphorylcholine)

f Poly­(acrylamide-acrylic acid)

g Polyethylene glycol.

h Cationic chitosan quaternary ammonium salt.

i Anionic sodium hyaluronate

j Poly­(ethylene glycol) diglycidylether

k Poly­(oxyalkylene amine)

l Microbial transglutaminase

m Polyvinylpyrrolidone.

n Polyimide.

o *N*,*N*-Dimethylformamide

p Polylactic acid.

q Polyethylene glycol diacrylate.

r *N*-isopropylacrylamide

s Polyacrylic acid.

t Acrylic acid.

u 2-Hydroxyethyl acrylate.

v Poly­(methyl methacrylate).

w Poly­(2-acrylamido-2-methylpropanesulfonic acid)

x Poly­(dodecyl glyceryl itaconate)

y Polyethylene amine.

z Poly­(vinyl alcohol).

As a distinctive mechanism of intermolecular interaction, entanglement significantly reinforces the mechanical properties of polymer networks, simultaneously avoiding the brittleness often induced by conventional crosslinking approaches. This property makes entanglement a key solution for enhancing the insufficient mechanical performance of hydrogels. Therefore, more and more research groups are actively exploring the mechanisms of entanglement and conducting experiments to evaluate its functional role in hydrogel development.^80,145^ Through the precise regulation of polymer entanglement and network configuration, significant theoretical advances can be realized, supported by experiments on various high-performance hydrogels.

Entanglement structures exert a profound impact on the functional properties of hydrogels. The presence of entanglements enhances the interactions between polymer chains, thereby increasing the modulus of the material. This effect is particularly pronounced in highly entangled hydrogels composed of high molecular weight polymers and low crosslinking density.^146–149^

Entanglement points serve as physical crosslinking points that increase the entropic elasticity of polymer chains, allowing for dynamic reconfiguration via entropy-driven restoration post-fracture, thereby enabling autonomous self-healing. Moreover, entanglements enhance the distribution of chain segments, resulting in a greater number of segments participating in the self-healing process at the fracture surface. Yan et al. conducted free radical polymerization of polyethylene glycol 480 methyl ether acrylate and *N*-acryloyl morpholine, increasing monomer concentration and lowering reaction temperature to promote polymer chain crowding, thereby generating a higher density of entanglement points.^114^ Introducing anisotropic structures into highly entangled polymers via pre-stretching can further elevate the tensile strength to 18,400 kPa. Most importantly, the polymer exhibits rapid self-healing capability, achieving a healing efficiency of 86.2% after only 30 seconds at room temperature.

The presence of entanglements also imparts more complex rheological behavior to the material under stress. The mechanical behavior of highly entangled hydrogels can be regulated through randomly distributed mobile entanglements.^149^ Cui et al. used in situ polymerization of acrylamide monomers within polyacrylamide nanogels, enabling the formation of densely entangled polymer chains at the nanoscale.^109^ This hydrogel demonstrates a remarkable swelling capacity, allowing it to retain high levels of water while maintaining a minimal polymer content. Despite containing water 98 wt % and exhibiting ultra-soft characteristics, these physically entangled hydrogels remain tough and stretchable due to entanglement cluster crosslinking interactions. Furthermore, these physically entangled hydrogels are capable of fully restoring their mechanical properties after lyophilization-rehydration treatment. In addition, entanglements enhance toughness and strength of materials by dissipating stress and preventing stress concentration. Hu et al. devised topologically constructed polymer networks with strong adhesive properties through topological structural design, simultaneously achieving enhanced ultimate elasticity and high toughness.^150^ Inspired by tendon structures, hard segments were incorporated into a soft substrate, by topological entanglements in the polymer network to realize strong adhesion. The prepared hydrogel demonstrates a 1.5 times greater elastic strain threshold, exceptional fracture toughness reaching 4.202  kJ m^–2^, modulus of 40 kPa, and significantly improved fatigue performance.

Meanwhile, the stress dissipation mechanism inherent to entanglement significantly reduces material hysteresis and enhances fatigue resistance. Zhao et al. utilized polyacrylamide hydrogel as the model material.^151^ By decreasing crosslinker content, the average polymer chain length is increased, thereby augmenting the number of entanglement points. Entanglement points in hydrogels serve as critical energy dissipation sites, particularly in the localized region near crack tips, where energy is dissipated through chain pull-out or chain delocalization of damage, significantly enhancing the fracture toughness of the hydrogel. The fatigue threshold of the entangled polymer network is 32  J m^–2^, while its fracture toughness exceeds this threshold by more than tenfold, reaching approximately 510  ±  48 J m^–2^, with hysteresis ratio from 5% to 10%. Suo and his colleagues prepared a transparent hydrogel by combining high molecular weight polymer chains, a small amount of water, and a photoinitiator.^129^ This equilibrium swollen, transparent hydrogel, with a polymer fraction of 20%, is capable of repeated knotting and twisting without damage, and exhibits outstanding anti-swelling capacity alongside high toughness (Figure 7). Furthermore, it combines nearly ideal elastic behavior with high tensile strength, excellent fatigue resistance, and low friction.

**Figure 7:**
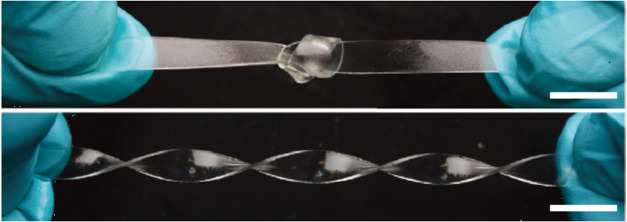
Hydrogel can be knotted and twisted. Reproduced from ref 129. with permission. Copyright 2022 Wiley.

### 7.2 Entanglement and Other Factors

Although individual entanglement structures significantly influence the mechanical properties and dynamic responses of hydrogels, their mechanisms are often constrained by the topological limitations of isolated networks. In fact, entanglements can synergistically couple with chemical crosslinking, hydrogen bonding, hydrophobic interactions, and other dynamic bonding mechanisms, or be combined with double network structures.^39,152–155^ The collaboration of multiple mechanisms not only strengthens the mechanical performance of the polymer network but also facilitates a superior balance between stiffness and toughness by diversifying energy dissipation pathways.^156,157^ Meanwhile, the integration of hierarchical network structures optimizes the structures of hydrogels, overcomes many bottlenecks and limitations in practical applications, and broadens their potential uses.^158,159^

Wang et al. proposed a novel high-toughness double network hydrogel composed of acrylamide as the monomer, *N*,*N*′-methylenebis­(acrylamide) as crosslinker, and 2-hydroxy-4′-(2-hydroxyethoxy)-2-methylpropiophenone as the initiator. By regulating the amount of 2-acrylamido-2-methylpropanesulfonic acid, the ratio between the primary and secondary networks was optimized to achieve superior mechanical performance.^91^ The hydrogel exhibits tensile strength of up to 3000 kPa and fracture toughness of 8.34 kJ m^–2^, demonstrating remarkable strain-hardening capability even at a high water content of 90 wt %. Compared with conventional double network hydrogels, this hydrogel exhibits minimal energy dissipation during stretching. Instead, it stores energy in the form of entropic loss, thereby achieving reversibility of up to 100%. This hydrogel endured 1000 cycles of stretching at 200% strain without degradation, demonstrating exceptional fatigue resistance. The highly entangled double network structure not only resolves the contradiction between high toughness and low hysteresis in hydrogels, but more importantly, offers new insights into the application of entangled structures in high performance hydrogels.

Lei et al. synthesized a stretchable and recoverable double-network hydrogel comprising a highly entangled network structure and a temperature-induced densely hydrogen bond network.^160^ Slidable entanglements and densely hydrogen bond function as efficient crosslinkers within the primary and secondary networks. Through efficient stress transmission along long polymer chains and energy dissipation via hydrogen bond clusters, the hydrogel achieves outstanding mechanical performance, including a stretchability of 999%, tensile strength of 820 kPa, and toughness of 4510 kJ m^–3^. Moreover, the hydrogel can fully recover to its original state after being subjected to puncture by needles and incisions by sharp surgical blades (Figure 8). It can also be demonstrated from the stress-time changes of hydrogel under 200 cycles at 100% strain and the stress variation is very small during loading-unloading cycles (Figure 9). Owing to the simple fabrication process and outstanding mechanical properties of this hydrogel, it can be molded into various shapes such as a clover, a pentagram, a teddy bear, and a heart (Figure 10a).

**Figure 8:**
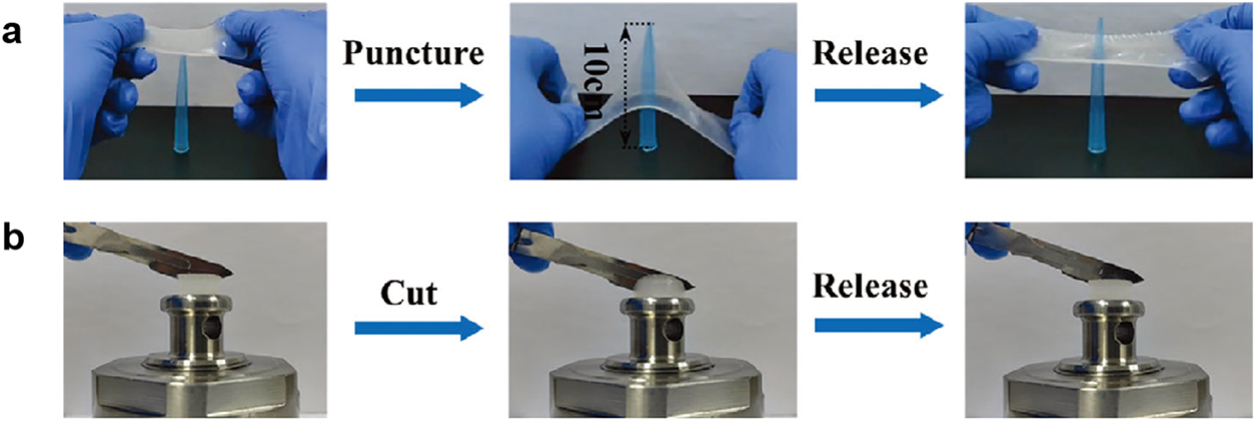
Entangled hydrogel exhibits excellent mechanical properties. (a) Resisting puncture by a needle. (b) Resisting incision by a blade. Reproduced from ref 160. with permission. Copyright 2025 Wiley.

**Figure 9:**
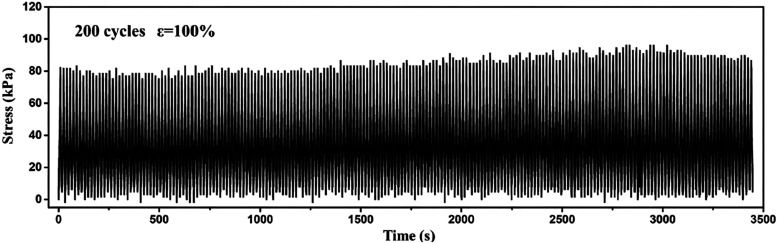
Tensile stress-time variation of entangled hydrogel under 200 consecutive cycles at 100% strain. Reproduced from ref 160. with permission. Copyright 2025 Wiley.

**Figure 10:**
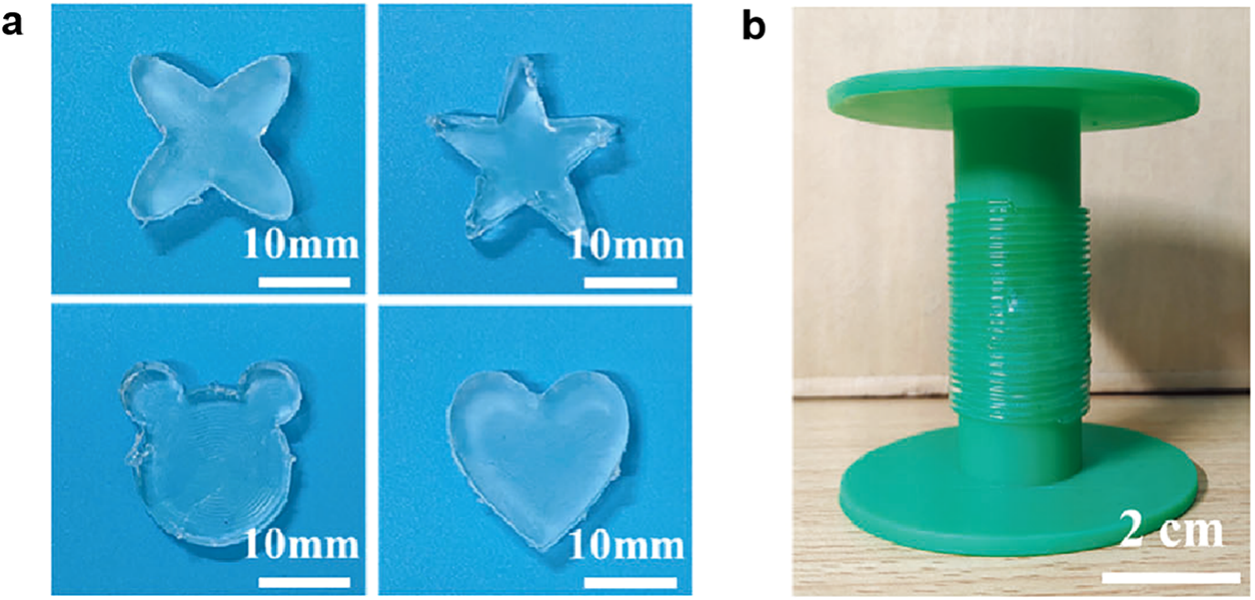
Entangled hydrogels exhibit excellent mechanical properties. (a) The hydrogel can be molded into different shapes. Reproduced from ref 160. with permission. Copyright 2025 Wiley. (b) The hydrogel fibers spun by the self-lubricating spinning strategy. Reproduced from ref 161. with permission. Copyright 2023 The Authors.

Yan and partners proposed a versatile self-lubricating spinning strategy for the continuous fabrication of hydrogel fibers from monomers, based on the hydrophobic mold induced regional heterogeneous polymerization.^161^ Benefiting from the universality of the self-lubricating spinning strategy, it can be combined with pre-gel design and post-treatment toughening processes to fabricate highly entangled polyacrylamide and ionic crosslinked poly­(acrylamide-*co*-acrylic acid) hydrogel fibers. The polyacrylamide hydrogel fibers exhibit excellent flexibility, allowing them to be coiled multiple times without fracturing (Figure 10b). The hydrogel fibers were able to lift 100 and 500 g weights (Figure 11a). Remarkably, the hydrogel fibers demonstrated outstanding mechanical strength (tensile stress exceeding 4000 kPa and tensile strain over 400%) following 120 days of swelling in environments with pH values between 3 and 9.

**Figure 11:**
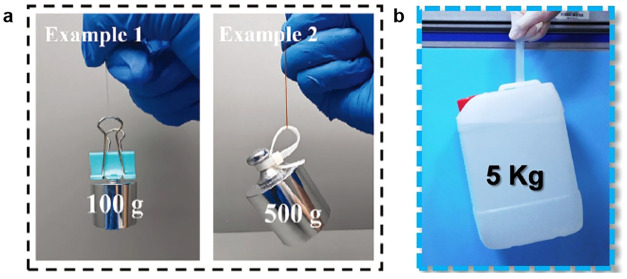
Entangled hydrogel exhibits excellent load-bearing capacity. (a) The polyacrylamide and ionic crosslinked poly­(acrylamide-*co*-acrylic acid) hydrogel fibers were able to lift 100 and 500 g weights. Reproduced from ref 162. with permission. Copyright 2023 The Authors. (b) Lifting a weight of 5 kg by the hydrogel. Reproduced from ref 162. with permission. Copyright 2022 American Chemical Society.

Shi et al. utilized polyelectrolyte microgels with disordered porous structures as confined reaction spaces for polymerization, facilitating entanglement between molecular chains.^162^ Through this approach, individual microgels act as microscale entanglement zones embedded in the hydrogel network, effectively reinforcing its mechanical performance. Researchers incorporated sodium polyacrylate hydrogel microspheres into polyacrylamide hydrogels via a straightforward compositing method, endowing the composite hydrogel with high tensile strength (260-990 kPa), sufficient to support a load of 5 kg (Figure 11b). Simultaneously, the hydrogel possesses high water content (63.40-84.74 wt %) and low initial modulus (22.61-112.45 kPa).

Wang et al. proposed a novel and simple method for constructing adhesive, self-healing, and highly stretchable double network hydrogels.^163^ The hydrogel is composed of a covalently crosslinked poly­(ethylene glycol) diacrylate network combined with a non-covalent network of extensively diffusive macromolecular polyethylene glycol chains. The hydrogel can self-heal within seconds and withstand tensile forces applied perpendicular to the cut interface (Figure 12). Owing to the diffusible polyethylene glycol chains that spontaneously penetrate and entangle with the substrate network, adhesion to substrates (including biological tissues) is immediate and repeatable, and the hydrogel exhibits remarkable adhesion to various surfaces (Figure 13a,b).

**Figure 12:**
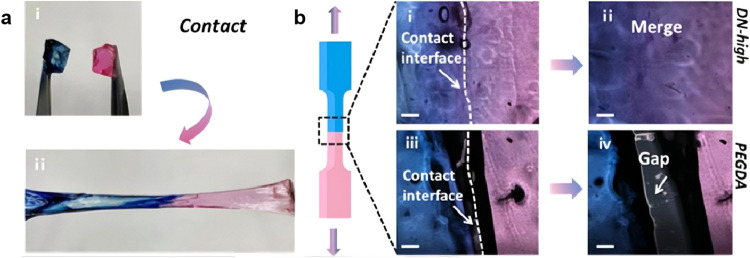
Self-healing properties of the entangled hydrogels. (a) Photographs showing the self-healing behavior of the entangled hydrogels. The hydrogel was cut into completely separate pieces and put back to close contact for 10 s followed by stretching. The hydrogels were painted by pink and blue dyes separately. (b) Optical microscopic images of fractured interfaces of (i), (ii) the entangled hydrogels in comparison to those of (iii), (iv) pure poly­(ethylene glycol) diacrylate gels. For entangled hydrogels, the fracture interface immediately merged after putting back into close contact. For poly­(ethylene glycol) diacrylate gels, the cutting edges remained to be clearly separated after putting back into contact. Scale bar: 50 μm. Reproduced from ref 163. with permission. Copyright 2019 American Chemical Society.

**Figure 13:**
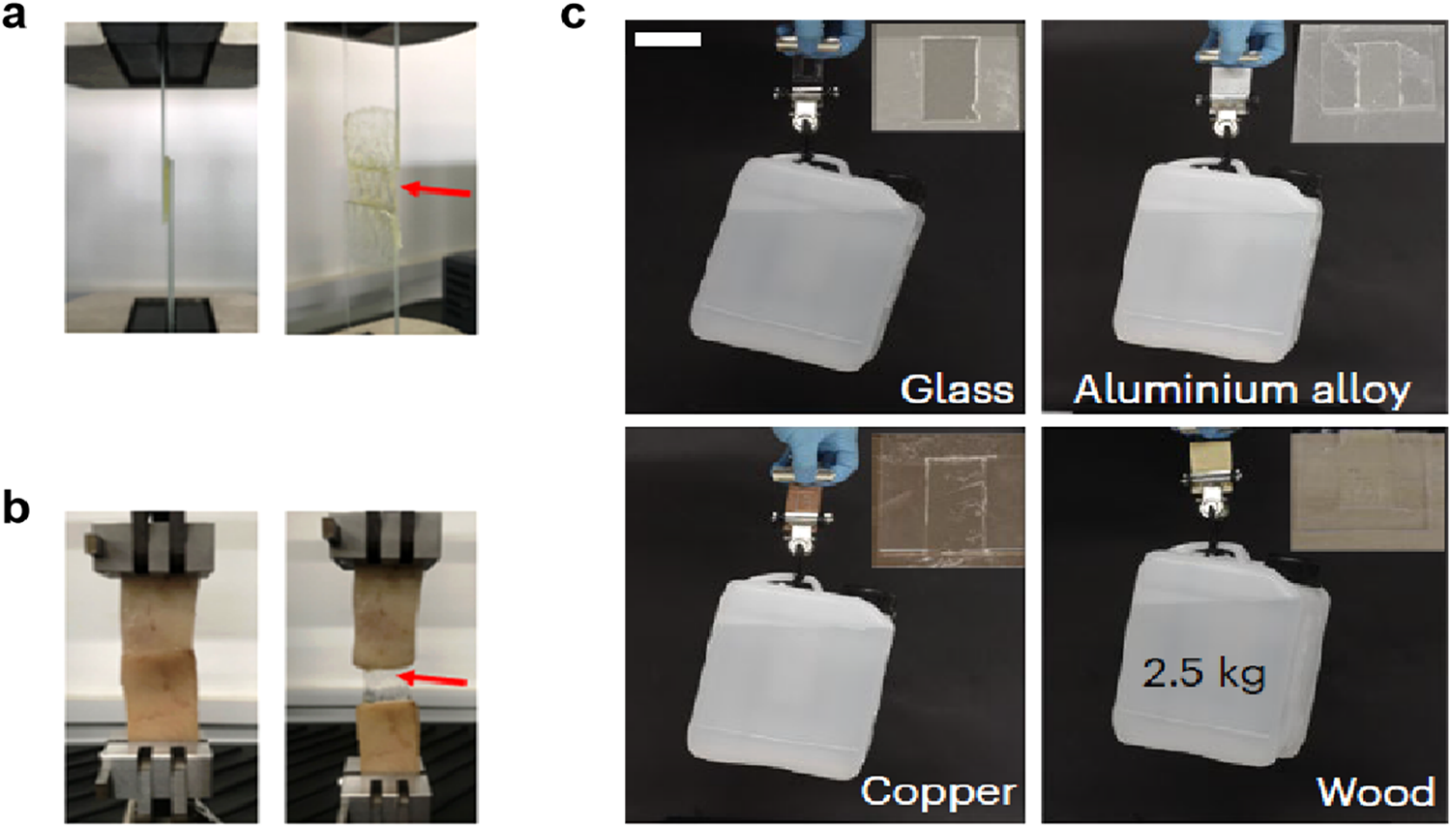
Adhesion properties of the entangled hydrogels. (a) Lap shear tests showing the adhesion behavior of the entangled hydrogels and commercial tissue sealants to glass slide and (b) porcine skin. Reproduced from ref 163. with permission. Copyright 2019 American Chemical Society. (c) Different substrates adhered by entangled hydrogels of 2 cm^2^ and thickness of 0.5 mm holding a weight of 2.5 kg. Scale bar: 5 cm. Reproduced from ref 40. with permission. Copyright 2019 The Authors.

Zhang et al. proposed a synthetic montmorillonite clay (hectorite) to construct coplanar liquid crystalline nanoconfinements, enabling the in situ polymerization of a highly entangled polyacrylamide network within the confined domains.^40^ A high concentration of acrylamide monomer (62 wt %) was introduced into the nanoconfined structure and polymerized under ultraviolet irradiation on to form a highly entangled polyacrylamide network, resulting in a nanoconfined polymer hydrogel. This hydrogel maintains a high modulus of 50,000 kPa while achieving up to 100% self-healing efficiency and demonstrating excellent adhesion across diverse substrates (Figure 13c).

He et al. in their research on toughening strategies for hydrogels, demonstrated the enhancement of mechanical properties by incorporating hydrophobic polymers.^164^ The hydrophobic polymer polylactic acid was introduced into a hydrophilic polyethylene glycol network, where entropy-driven compatibility promoted tight entanglement between the hydrophobic chains and the hydrophilic network. The hydrophobic chains aggregate in water to form semi-crystalline domains that reinforce the network’s stiffness, while the entanglements between chains confer the hydrogel with enhanced toughness and fracture resistance. Through uniaxial stretching experiments, the amphiphilic hydrogels exhibited modulus 150–400 kPa and toughness ranging from 1200-3900 kJ m^–3^, markedly higher than those of pure poly­(ethylene glycol) diacrylate hydrogels. Experimental data demonstrate that the hydrogel exhibits not only stiffness, toughness, and durability under high swelling ratios, but also tunable mechanical properties through regulation of polylactic acid content and crosslinking conditions.

Inspired by polymer entanglement, Long et al. reported a simple approach to incorporating hydroxypropyl cellulose fibers into various smart hydrogel substrates systems through entanglement.^138^ By compositing hydroxypropyl cellulose with thermos responsive poly­(*N*-isopropylacrylamide), the fibers become entangled with the substrate and form hydrogen bonds, in contrast to conventional smart hydrogels where polymer networks are interconnected through chemical crosslinking. Consequently, the combined presence of entanglements and hydrogen bonds enhances the mechanical properties while preserving the responsive functionalities of the polymer network. The mechanical strength of the hydrogel increases with the rising content of hydroxypropyl cellulose. By compounding with hydroxypropyl cellulose, the composite hydrogel shows ∼10–22 times improvement in modulus and toughness compared with pure hydrogel. More importantly, compared to the control group, this composite material exhibits enhanced smart functionalities, including superior cyclic stability, accelerated response rates, and heightened resistance to drying and freezing.

Liu et al. introduced a dissipative energy molecular engineering approach that leverages the molecular-level synergy between strong and weak interactions: strong interactions such as metal coordination bonds impart mechanical robustness, whereas weak interactions like hydrogen bonds alleviate material yielding. Subsequently, dense entanglements were further incorporated into the network, with these dense entanglements ensuring rapid recovery through entropic elasticity.^106^ This hydrogel demonstrated a remarkable synergy of elevated strength (1200 kPa) and superior toughness (2580 kJ m^–3^), alongside rapid elastic recovery on the order of seconds. The essence of dissipative molecular engineering is the energy dissipation within polymer networks. After 20 cycles of stretching-relaxation, the stretched gel was immersed in water for 5 min, instantly restoring to its original state and enabling subsequent uniaxial tensile testing.

## 8 Applications

Entangled hydrogels, distinguished by their high water content, remarkable resilience, impressive toughness, excellent self-recovery, and superior mechanical properties, exhibit advantages over conventional hydrogels in numerous domains, thus showing great potential for application in various fields.^176,177^ For instance, in tissue engineering, cell culture requires an environment with high water content to facilitate nutrient diffusion, while also demanding the gel to provide adequate binding sites to support cell adhesion and proliferation. The high-permeability network and tunable mechanical properties of entangled hydrogels precisely meet these needs, making them an ideal choice for 3D cell culture scaffolds. In the realm of flexible sensors, high resilience is crucial; however, conventional methods for enhancing mechanical strength, such as increasing crosslinking density or adding rigid fillers, often compromise the material’s ability to recover elastically. The dynamic reversible network structure of entangled hydrogels allows them to swiftly recover after enduring repeated deformations, ensuring the sensors’ long-term stability and signal reliability. Moreover, these hydrogels possess expansive potential across domains like soft robotics, drug delivery, and electronic skin technologies.^178,179^ The adjustability of their properties and adaptability of their structures offer novel insights into the creation of future functionalized materials.^119^

### 8.1 Biomedical Field

Hydrogel applications in the biomedical field require not only robust mechanical properties but also exceptional biocompatibility.^180,181^ Conventional chemically crosslinked hydrogels, while possessing good mechanical properties, often require the use of crosslinking agents during their preparation.^182^ These agents can leave behind toxic residues, compromising the hydrogels’ biocompatibility.^183^ Entangled hydrogels, which primarily use physical entanglement as their crosslinking points, largely avoid the issue of toxic residue. These hydrogels not only possess excellent mechanical properties but also better retain the natural biological characteristics of the polymers, resulting in improved biocompatibility.^184^ This makes them particularly suitable for sensitive tissues or long-term implants.^122,185–188^ Because entangled hydrogels are highly compatible with biocompatible polymers, they are considered ideal materials for soft tissue repair.

Xuefeng Li and colleagues proposed a type of polyelectrolyte complex hydrogel that is highly entangled with natural polysaccharides.^130^ Through experiments conducted on mice, it was demonstrated that this hydrogel can significantly accelerate wound healing, achieving a wound healing rate of 94.32% ± 0.99% by post-operative day 14 (Figure 14). Furthermore, new blood vessels and hair follicles, along with scar-free regenerated epidermis, were observed only in the wound tissue treated with this hydrogel. These findings strongly suggest that this composite hydrogel material has great potential as a wound dressing for scar-free skin regeneration.^189–191^

**Figure 14:**
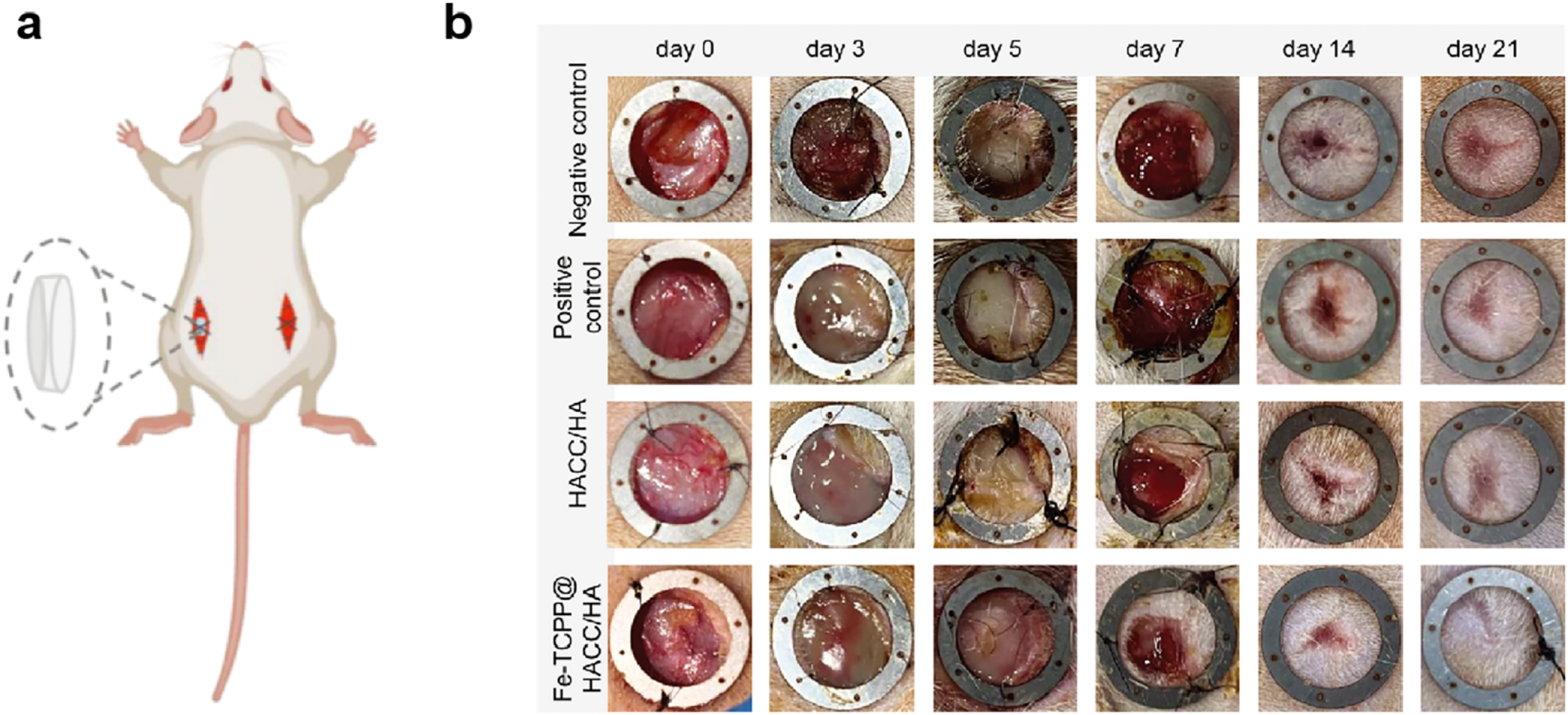
Entangled hydrogel exhibits excellent healing properties for wounds. (a) Schematic illustration of open wound treatment with hydrogels using Sprague Dawley rat model and (b) photographs of wounds treated with different hydrogels in a 21-day period of time. Reproduced from ref 130. with permission. Copyright 2024 Wiley.

Entangled hydrogels demonstrate exceptional efficacy in repairing surface skin injuries and hold great promise for applications in repairing internal bodily damage.^192,193^ Articular cartilage, with its elasticity and compressibility, effectively cushions and absorbs shock, reducing direct bone friction during movement, thereby safeguarding the joint structures from harm. In the long term, the health of articular cartilage directly influences joint functionality. Thus, research into treatments for cartilage damage is particularly crucial. Tissue engineering through the use of artificial substitutes for replacement and regeneration presents an effective approach.^194^ To date, various scaffolds for artificial substitutes have been developed to withstand loads, control drug release, and mimic the natural physiological environment of cells.^195^ Hydrogels, due to their excellent biocompatibility and structural stability, are considered ideal scaffold materials for articular cartilage and show significant potential in cartilage replacement applications.^196–198^

Inspired by the entangled network of collagen and proteoglycans that imparts high stiffness and toughness to articular cartilage, Hongbin Li and colleagues developed an entangled elastin-based N-DC (Native-state, Denatured-state Crosslinked) hydrogel designed to simulate cartilage for biomedical applications.^165^ By employing a denaturing crosslinking method to prepare the artificially designed ferritin-like protein (FL) repetitive unit (FL)­8, the entangled network is formed, which is then partially refolded to restore the natural structure of FL. The resulting hydrogel integrates the seemingly contradictory properties of high stiffness, high toughness, and rapid recovery. The Young’s modulus of the hydrogel reaches 700 kPa, with a compressive strength of 68000 kPa and a compression modulus of 1700 kPa. Additionally, the hydrogel exhibits excellent fatigue resistance during tensile and compressive cycles, effectively transforming soft protein biomaterials into a hard and tough material with mechanical properties approaching those of cartilage. In vitro, the hydrogel supports cell adhesion, proliferation, and differentiation; in vivo, it was used to repair osteochondral defects in a rabbit model, demonstrating significant cartilage and bone tissue regeneration capabilities. The hydrogel’s combination of high stiffness and toughness, rapid recovery, fatigue resistance, and excellent biocompatibility makes it an ideal material for cartilage simulation, providing a novel solution for osteochondral defect repair.

Highly entangled hydrogels become a core focus in 3D printing research.^199,200^ Hydrogel biomaterial scaffolds play an important role in simulating cartilage and also provided a foundation for modeling the three-dimensional structure of whole organs.^201,202^

Jason A. Burdick and partners proposed the CLEAR (continuous-curing after light exposure aided by redox initiation) printing strategy, which enables high conversion rates and dense entanglements in a single step at room temperature, allowing 3D printing of single-network hydrogels with both high stiffness and toughness.^203^ By introducing carboxyl groups and synergizing with bridging polymers such as chitosan, the hydrogels can form physical-mechanical interlocks with wet biological tissues, achieving high interfacial adhesion toughness. This supports complex topologies, spatially programmable adhesion zones, and hydrogel-elastomer hybrid structures, meeting medical requirements such as suturability, rehydration after dehydration, and cell compatibility, providing a new platform for next-generation tissue adhesives and monitoring devices.

Lv and colleagues developed a poly­(vinylpyrrolidone)/polyimide entangled hydrogel with added hydrophobic polymer.^169^ The viscosity of the hydrogel solution is sufficiently high to ensure that the extruded filaments maintain a certain shape, and by adjusting the mass ratio of poly­(vinylpyrrolidone) and polyimide, the viscosity and rheological properties of the solution can be controlled. This makes it suitable for use as an ink material for 3D printing. This multi-material structure can provide more suitable mechanical properties, better simulating the structure of natural tissues. When NCTC clone 929 cells were seeded onto the printed poly­(vinylpyrrolidone)/polyimide scaffold, they exhibited a high survival rate; primary human chondrocytes cultured in the hydrogel solution also maintained good viability and proliferation ability. This has promising applications in tissue engineering.

Additionally, the entangled hydrogel can serve as a cell carrier, embedding stem cells or growth factors to further enhance tissue repair effectiveness.^204,205^ The swelling-shrinking property of the entangled hydrogel offers unique advantages in drug delivery systems.^206,207^ Drugs can be encapsulated within the network structure of the hydrogel, allowing for slow release by adjusting the composition and structure of the hydrogel. For instance, in the treatment of chronic diseases, the entangled hydrogel can act as a long-lasting drug carrier, reducing the frequency of medication administration for patients. The release rate can be precisely controlled by altering factors such as the hydrogel’s crosslinking density and degree of swelling.^208,209^

### 8.2 Sensor

As a critical hub for detecting and converting physical or chemical quantities, sensors need to possess high sensitivity and accuracy, allowing them to respond quickly and accurately to changes in the measured parameters. For example, an anti-swelling zwitterionic nanocomposite hydrogel was used as an underwater sensor,^210^ a cellulose-based ionic-conductive hydrogel was developed for flexible electronics,^211^ and 4D-printed thermochromic hydrogel wearables have been demonstrated for temperature and UV monitoring.^212,213^ This requires sensing materials to have excellent mechanical strength, a broader operating range, and good fatigue resistance. If used for human monitoring, the materials must also be biocompatible and flexible enough to ensure they don’t hinder human movement while providing rapid response. Based on these requirements, entangled hydrogels undoubtedly become a viable alternative to inorganic materials for developing wearable sensors.^214^ By introducing conductive fillers into the hydrogel, it is possible to create a strain-sensitive hydrogel.^215^ When the hydrogel is subjected to external force, its conductivity changes, allowing real-time monitoring of the force through detecting changes in conductivity.

Jia et al. designed stretchable strain sensors using highly entangled polyacrylamide hydrogels with high modulus and low hysteresis.^216^ The high modulus ensures robust performance, allowing detection of elongation with a resolution of up to 0.2% under small deformations. The sensor can measure the elongation of elastomers within a range of 200%. The change in relative resistance maintains low hysteresis and is repeatable over 1000 testing cycles. The hydrogel sensor, with water-retaining encapsulation, exhibits long-term electrical stability.

Chen and colleagues constructed highly stretchable supramolecular polyacrylic acid/polyaniline hydrogels with entangled networks by doping polyaniline with acrylic acid monomers.^170^ The high-density electrostatic interactions between polyacrylic acid and polyaniline chains act as dynamic bonds, inducing entanglement. The conductivity of polyacrylic acid/polyaniline hydrogels is one order of magnitude higher than that of pure polyacrylic acid hydrogels, providing faster electron transfer rates. Additionally, polyacrylic acid/polyaniline hydrogels exhibit exceptional stretchability (2830%), high breaking strength (120 kPa), and rapid self-healing capabilities. Furthermore, polyacrylic acid/polyaniline hydrogels have high strain sensitivity and rapid response times. Their excellent mechanical properties ensure stable and repeatable response signals during cyclic testing, demonstrating good stability. Sensors made from highly entangled hydrogels can achieve high resolution even under minimal deformation. This means that in practical applications, not only large-scale movements (such as running, jumping, squatting, etc.) can be monitored, but small physiological activities can also be accurately detected and recorded (Figure 15), expanding the working range and sensitivity of the hydrogel and broadening the application scenarios for sensors.

**Figure 15:**
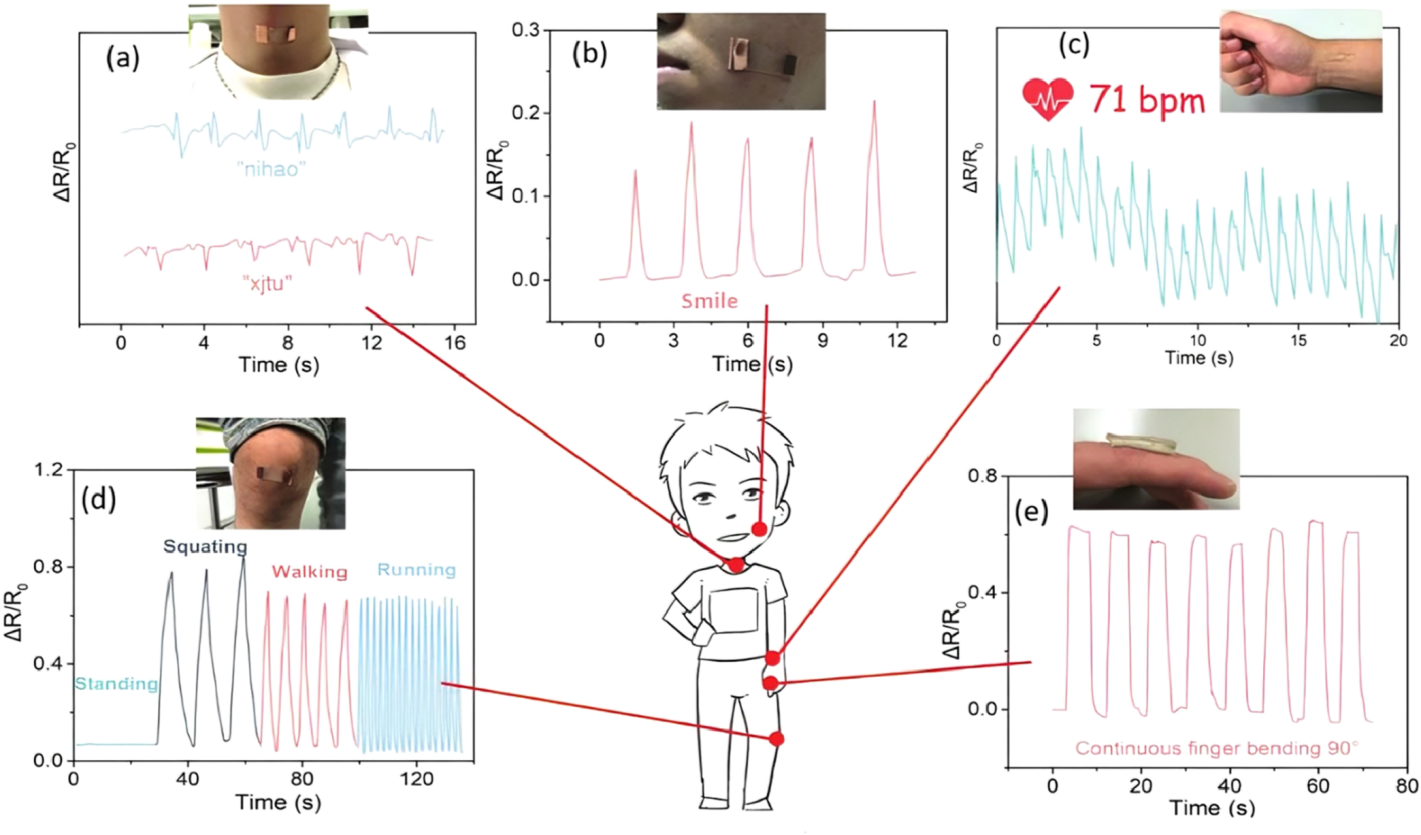
Real-time sensing performance of the entangled hydrogel sensor. (a) Throat vocalization, (b) cheek, (c) wrist, (d) knee, and (e) finger. Reproduced from ref 170. with permission. Copyright 2022 The Authors.

### 8.3 Environmental Field

Entangled hydrogels can adsorb pollutants such as heavy metal ions and organic substances from water and soil through their network structure.^217,218^ For instance, Han et al. reported a gradient P­(AA-AM-NH_2_-β-CD) hydrogel reinforced via Hofmeister effects for autonomous enrichment of ultra-low-concentration Sb­(III).^219^ Their adsorption capacity can be optimized by adjusting the composition and surface properties of the hydrogel. For example, when treating wastewater containing heavy metal ions, specific functional groups can be introduced into the hydrogel to achieve selective adsorption of these ions.^220^ Furthermore, entangled hydrogels can perform adsorption-desorption cycles through their swelling and shrinking properties, which improves the lifespan of the adsorbent. In water treatment processes, filtration is an important technique. Entangled hydrogels can be used as filtration materials, capturing suspended particles and microorganisms in water through their network structure. The pore structure can be precisely controlled by adjusting the hydrogel composition and preparation method, enabling filtration of particles of different sizes. Additionally, the flexibility of entangled hydrogels allows them to withstand a certain amount of pressure during filtration, ensuring filtration efficiency.

Tian and colleagues successfully addressed the contradiction between swelling and adsorption in hydrogel adsorbents by introducing hydrogels with rich carboxyl functional groups that have high entanglement.^168^ The hydrogels demonstrated extremely high adsorption capacities for cationic pollutants such as methylene blue and lead ions, reaching 1335.8 mg/g and 375.4 mg/g, respectively, which are far higher than those of conventional highly crosslinked hydrogels (Figure 16). Moreover, their adsorption rates are fast; for example, methylene blue reaches adsorption equilibrium in about 50 min, with an adsorption rate 13.6 times faster than that of highly crosslinked hydrogels. The addition of entanglement allows the hydrogel to swell significantly under alkaline conditions, increasing the exposure of adsorption sites and the diffusion of pollutants; under acidic conditions, it effectively dehydrates. Through these swelling and shrinking properties, entangled hydrogels successfully achieve adsorption-desorption cycles, facilitating regeneration and reuse, thereby enhancing the lifespan of the adsorbent. Even after multiple adsorption and desorption cycles, they maintain high adsorption capacity, showing good adsorption-desorption cycle capability and stability. Additionally, highly entangled hydrogels exhibit high selectivity in adsorption, offering great potential for applications in smart separation.

**Figure 16:**
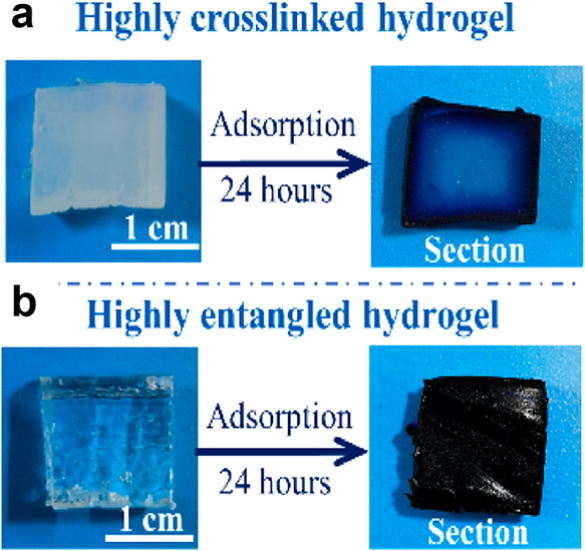
Adsorption property of the entangled hydrogel. (a) Highly crosslinked hydrogel and (b) highly entangled hydrogel and the corresponding cross-section images after full adsorption of methylene blue. Reproduced from ref 168. with permission. Copyright 2022 Elsevier.

Yu and colleagues developed an innovative injection-driven filtration system.^221^ This system combines a fully biodegradable biomass-based nanofiber hydrogel membrane with a syringe for the specific purpose of removing ultrafine suspended solids, achieving portable and sustainable water purification. The hydrogel membrane features a densely packed and entangled nanofiber network, with the ability to retain ultrafine suspended solids with efficiency close to 100%, and a cutoff size of about 10 nm, significantly surpassing that of commercial filter paper and microporous filter membranes. During the water purification process, this filtration system achieves a water flux as high as 90.6 g per square centimeter per hour, which is 7.2 times greater than that of commercial polycarbonate ultrafiltration membranes. Additionally, the filtration system is not only cost-effective but also highly scalable and reusable, while maintaining environmental friendliness and sustainability.

### 8.4 Energy Field

Ideal materials for application in the energy storage sector should possess high energy density, excellent cycling stability, high safety, high adaptability, and low interfacial impedance.^222,223^ Conventional materials face challenges such as insufficient mechanical flexibility, high interfacial impedance, poor safety, and difficulty in adapting to complex deformation environments. Entangled hydrogels perfectly meet these requirements due to their unique entangled network structure.^224^ Their high ionic conductivity and adjustable porous structure ensure efficient ion transport, significantly enhancing charge and discharge efficiency.^225^ The superior flexibility and stretchability of the material allow devices to operate stably under bending, folding, and even stretching conditions, making them suitable for wearable devices. Additionally, the dual solid-liquid phase characteristics of hydrogels can inhibit dendrite growth and achieve self-healing through dynamic bonds, extending their cycling lifespan. Moreover, their high water content and biocompatibility provide a safe solution for implantable energy storage, and modification with components such as antifreezing and moisturizing agents can further expand the material’s application scenarios to meet extreme environmental requirements.

Baohua Li and colleagues developed a hydrogel electrolyte with a harmonious combination of mechanical strength, ionic conductivity, and interfacial adaptability by constructing a hydrogel based on a highly entangled network.^172^ The hydrogel exhibits exceptional mechanical properties, with a tensile strength of 6800 kPa and an elongation at break of 483.3%, offering good flexibility, which allows it to be molded into various shapes suitable for mass production and soft battery assembly. Its ionic conductivity reaches 3.68 mS/cm at 20°C, demonstrating excellent temperature adaptability. Moreover, the hydrogel electrolyte regulates zinc electrodes by preventing dendrite growth; its surface carries negative charges that effectively adsorb Zn^2+^ions, promoting the desolvation process of Zn^2+^. The hydrogel maintains excellent stability and capacity retention even after prolonged multiple cycles, providing new insights and methods for the future development of high-performance aqueous zinc metal batteries.

Flexible aqueous zinc-ion batteries have become important candidate materials for next-generation safe energy storage and wearable electronic devices due to their high safety, exceptional flexibility, low cost, and simple manufacturing processes. However, zinc-ion batteries assembled with conventional hydrogel electrolytes have drawbacks such as poor rate performance, slow kinetics, and low ionic conductivity, making it difficult to achieve applications that require fast charging and discharging.

Hong and colleagues applied the design of highly entangled hydrogel electrolytes to aqueous zinc-ion batteries.^225^ They enhanced the extensibility of the hydrogel network structure and the degree of entanglement between chain structures, reduced the obstruction caused by crosslinking between chain structures, and constructed fast ion transport channels. Through the design of highly entangled hydrogel electrolytes, they addressed the weaknesses of conventional hydrogel electrolytes. The synthesized highly entangled polyacrylamide hydrogel significantly improves the ion transport and mechanical stability of the electrolyte, exhibiting high ionic conductivity, strong elastic modulus, and a long shelf life. The assembled battery maintains high rate and long cycle performance, and can perform charge and discharge cycles at the highest current density (35A/g). Additionally, this hydrogel electrolyte also supports proton batteries to conduct fast charge and discharge at a maximum current density of 80 A/g.

Daytime passive cooling technology has garnered significant attention due to its lack of external energy consumption and environmental friendliness.^226^ However, its application still faces challenges such as the difficulty in simultaneously optimizing reflectivity and emissivity, insufficient material durability, and the trade-off between evaporation cooling and nighttime water replenishment. Entangled hydrogels, through their unique polymer network structure, offer both high solar reflectivity and efficient mid-infrared emissivity. Meanwhile, the dynamic crosslinked entangled structure within provides the material with excellent toughness and self-healing capabilities, allowing it to withstand outdoor UV, temperature variations, and mechanical wear over the long term. Additionally, the porous hydrophilic nature of the hydrogel enables it to assist in cooling through water evaporation, and it can maintain stable radiative cooling performance even in dry environments, adapting to different climate conditions. Compared to conventional passive cooling materials, entangled hydrogels not only achieve a balance between optical performance and mechanical stability but also overcome the efficiency bottleneck of single-mode cooling through an evaporation-radiation synergistic cooling effect, providing a low-cost, long-lasting solution for sustainable daytime cooling.

Zhu and colleagues addressed the contradiction between evaporation cooling and nighttime water replenishment in daytime passive cooling technology by introducing a highly entangled structure and hygroscopic agents to prepare zirconium dioxide@polyacrylamide hydrogels.^166^ Compared to conventional hydrogels, the hydrogel exhibits an 18-fold increase in tensile strength and a 9-fold increase in compressive strength. This high-strength hydrogel maintains good mechanical performance even after complete water absorption. Additionally, the hydrogel has a solar reflectivity of 0.90 and a mid-infrared emissivity of 0.88 in the atmospheric window, enabling effective daytime radiative cooling. By incorporating lithium bromide into the hydrogel, its hygroscopicity is significantly enhanced, allowing it to absorb moisture from the atmosphere under high humidity conditions at night. This process enables cooling through evaporation during the day, achieving a hygroscopic-evaporation cycle that allows the hydrogel to provide sustainable cooling without requiring an external water source. This presents a new approach to developing sustainable daytime passive cooling technologies.

## 9 Conclusion and Prospect

### 9.1 Conclusion

Entangled hydrogels provide a clear and physically grounded route to addressing the long-standing stiffness-toughness contradiction in conventional hydrogels. Instead of relying solely on a high covalent crosslinking density—which often raises modulus at the expense of extensibility and fatigue resistance—entanglement-based topological design enables load sharing and recoverable dissipation while maintaining swelling and deformation capability. From the viewpoint of polymer physics, the tube model and reptation framework offer a useful language for describing how these constraints limit chain motion and store elastic energy. In this context, the entanglement molecular weight (*M_e_*) and related topological parameters become key microscopic descriptors for interpreting macroscopic mechanical responses, including strength, hysteresis, recovery, and fatigue behavior.

Across the diverse preparation routes summarized in this work, such as high concentration monomer polymerization, kneading-annealing processing, the underlying logic is remarkably consistent. Each method aims to increase effective chain overlap and interpenetration, or to promote conformational organization followed by physical interlocking, so that entanglements substantially outnumber permanent crosslinking. High concentration monomer creates a crowded environment that favors chain interweaving during polymerization; kneading-annealing homogenizes dense polymer “doughs” and locks in entanglements prior to sparse crosslinking and swelling. Although the chemistry and processing windows differ, the shared goal is to build a high-density entanglement network that can bear load, dissipate energy in a controlled manner, and recover after deformation.

A central message is that entanglements are invisible topological features, and no single characterization method can quantify them reliably on its own. Macroscopic mechanical tests (tensile, compression, rheology) provide essential performance metrics and indirect signatures of entanglement-dominated elasticity and dissipation, but they do not uniquely identify topology. Scattering and imaging techniques (e.g., SAXS/SANS and polarization-based methods) help reveal deformation-induced anisotropy and chain orientation across length scales, while spectroscopic approaches (such as NMR-related probes and dielectric relaxation) access the time-dependent constraints on segmental motion. Only by combining these complementary tools can one build a credible structure-property picture that links entanglement density and dynamics to measurable mechanical behavior.

Importantly, the value of entangled hydrogels is not abstract. Their combination of high modulus, high toughness, low hysteresis, and strong fatigue tolerance makes them attractive for demanding applications where conventional hydrogels fail: soft tissue substitutes and cartilage-mimicking scaffolds that must sustain repetitive loading; flexible sensors and wearable devices that require stable signals over many cycles; and energy platforms where both function and long-term integrity matter (e.g., hydrogel electrolytes under deformation, or outdoor materials for passive cooling that must withstand UV exposure, temperature cycling, and wear). Overall, entanglement should be viewed not simply as a mechanical enhancement trick, but as a practical topology design principle that connects polymer physics to real device-level performance.

### 9.2 Prospect

To move from impressive demonstrations to broadly usable materials, future research should focus less on conceptual demonstrations and more on three concrete questions: How can we make entangled networks more predictable, more durable, and more controllable. Across the field, several recurring bottlenecks limit the translation of entanglement-rich hydrogels: (i) difficulty isolating entanglement-controlled mechanics from coexisting physical associations, (ii) lack of quantitative metrics for trapped constraints after swelling/aging, and (iii) insufficient fatigue/creep/environmental durability tests. Potential solutions include standardized reporting of preparation and swelling history, coupling mechanical tests with in situ structural probes to track constraint persistence, and integrating multiscale simulations and data-driven models to build quantitative structure–topology–property mappings.

(1) Polymer physics in swollen networks: solvent effects and non-equilibrium behavior
  Most classical entanglement theories were developed for polymer melts or concentrated solutions, whereas hydrogels operate in a swollen state where water (or mixed solvents) strongly alters chain conformation and mobility. A priority is therefore to adapt or extend entanglement physics to swollen polymer networks.
  First, the thermodynamic role of the dispersing medium needs deeper study. Solvent quality changes chain dimensions and effective overlap, which can shift the entanglement threshold and modify the stability of the entanglement network after swelling. Second, solvent-dependent friction and local segmental mobility can alter the effective tube diameter and relaxation times, directly influencing viscoelasticity, hysteresis, and recovery. Third, under large deformation in a highly swollen state, entanglement points are not strictly fixed: they may slide, rearrange, or progressively disentangle. Understanding the kinetics of sliding and disentanglement, especially under cyclic loading, will be essential for building non-equilibrium models that are genuinely applicable to hydrogels rather than borrowed from melt-state assumptions. A practical outcome of this direction would be physics-based guidelines that relate swelling ratio and solvent conditions to retention (or loss) of entanglement-controlled mechanics.
  To clarify what entanglement constraints physically mean in swollen networks, future studies should combine mechanical tests with structure-sensitive probes under controlled swelling histories (e.g., coupling rheology or tensile tests with in situ scattering, and using spectroscopic/relaxation measurements to quantify time-scale-dependent constraint persistence). On the modeling side, multiscale simulations can be used to explicitly quantify topological constraints (e.g., via primitive-path-based metrics) in the preparation state and after swelling, thereby linking ‘trapped entanglement’ statistics to macroscopic signatures such as hysteresis, recovery, and fatigue threshold. Establishing such structure-topology-property mapping will be essential for converting entanglement from a qualitative concept to a predictable design parameter.
(2) Durability and environmental stability: toward all-environment evaluation standards
  Many reports still emphasize short-term, extreme metrics (ultimate stretch, peak strength) while real applications depend on long-term stability. For entangled hydrogels, the next step is to build a durability framework that reflects realistic service conditions.
  A shift is needed from high strength to high stability. Besides fracture toughness, key targets include creep resistance, fatigue crack growth behavior, aging under continuous hydration, and wear under repeated contact or friction. Environmental coupling is equally important. Outdoor or device-facing uses may involve UV radiation, temperature cycling, and exposure to salt/acid/base, all of which can change polymer mobility, weaken secondary interactions, and accelerate network evolution. Another underexplored but decisive factor is dehydration-rehydration cycling: many hydrogels experience water loss, collapse, and reswelling. Whether entanglement topology undergoes irreversible rearrangement during these cycles will largely determine reusability and lifetime. Finally, the field would benefit from standardized testing protocols tailored to entanglement-dominated gels, so performance claims are not reduced to one beautiful stress-strain curve, but supported by comparable fatigue, creep, and environmental aging datasets.
(3) Data-driven prediction and simulation: quantitative structure-property mapping

Despite clear progress, many formulations are still developed by trial and error because it remains difficult to predict macroscopic properties from microscopic topology. A realistic and impactful prospect is to construct quantitative mapping between entanglement density and mechanical performance.

On the simulation side, molecular dynamics and coarse-grained methods (including slip-link or network reorganization models) can be used to connect chain length distribution, concentration history, and processing conditions to M_e_, tube-scale parameters, and time-dependent relaxation behavior. On the data side, integrating experimental datasets-rheological spectra, swelling ratios, fatigue metrics, and structural signatures-would enable predictive models that estimate target ranges of modulus, hysteresis, and durability from controllable inputs (monomer type, concentration, polymerization/annealing conditions, and crosslinking strategy). The longer-term aim is to move from explaining to designing: using simulation and data-driven tools to propose topological programming strategies, such as spatial gradients in entanglement density or tailored chain-length distributions, to meet application-specific requirements with fewer experimental iterations.

### 9.3 Concluding Remarks

It is important to be sober about the boundaries of the entanglement strategy. Entanglements are not a universal cure: creating highly entangled networks often increases precursor viscosity, complicates mixing and degassing, and can limit processing speed or scalability. Dense networks may also slow mass transport and response kinetics in applications that require fast diffusion or rapid actuation. Moreover, swelling and environmental exposure can gradually weaken the effective topological constraints, meaning that high entanglement does not automatically guarantee long service life without careful design and validation.

For these reasons, entanglement should be positioned as a general topology design tool rather than a single material category. Its real strength lies in compatibility: entanglements can work together with sparse covalent crosslinking, supramolecular interactions, nanocomposite reinforcement, or hierarchical architectures to balance robustness, functionality, and manufacturability. Looking forward, the most meaningful vision is not merely to make hydrogels stronger, but to make their topology programmable, so that polymer scientists can tune entanglement formation, distribution, and dynamics with the same deliberateness used to design macroscopic structures. Achieving this level of control will help entangled hydrogels transition from compelling laboratory materials to the next generation of reliable soft matter for practical and demanding environments.
